# A High-Throughput Screen Identifies 2,9-Diazaspiro[5.5]Undecanes as Inducers of the Endoplasmic Reticulum Stress Response with Cytotoxic Activity in 3D Glioma Cell Models

**DOI:** 10.1371/journal.pone.0161486

**Published:** 2016-08-29

**Authors:** Natalia J. Martinez, Ganesha Rai, Adam Yasgar, Wendy A. Lea, Hongmao Sun, Yuhong Wang, Diane K. Luci, Shyh-Ming Yang, Kana Nishihara, Shunichi Takeda, Mohiuddin Sagor, Irina Earnshaw, Tetsuya Okada, Kazutoshi Mori, Kelli Wilson, Gregory J. Riggins, Menghang Xia, Maurizio Grimaldi, Ajit Jadhav, David J. Maloney, Anton Simeonov

**Affiliations:** 1 National Center for Advancing Translational Sciences, National Institutes of Health, Rockville, MD, 20850, United States of America; 2 Department of Radiation Genetics, Graduate School of Medicine, Kyoto University, Yoshidakonoe, Sakyo, Kyoto 606–8501, Japan; 3 Department of Biophysics, Graduate School of Science, Kyoto University, Kitashirakawa-Oiwake, Sakyo, Kyoto 606–8502, Japan; 4 Department of Neurosurgery, John Hopkins University, Baltimore, MD 21231, United States of America; 5 Laboratory of Neuropharmacology, Department of Biochemistry and Molecular Biology, Southern Research Institute, Birmingham, AL 35205, United States of America; Toho Daigaku, JAPAN

## Abstract

The endoplasmic reticulum (ER) is involved in Ca^2+^ signaling and protein folding. ER Ca^2+^ depletion and accumulation of unfolded proteins activate the molecular chaperone GRP78 (glucose-regulated protein 78) which in turn triggers the ER stress response (ERSR) pathway aimed to restore ER homeostasis. Failure to adapt to stress, however, results in apoptosis. We and others have shown that malignant cells are more susceptible to ERSR-induced apoptosis than their normal counterparts, implicating the ERSR as a potential target for cancer therapeutics. Predicated on these findings, we developed an assay that uses a GRP78 biosensor to identify small molecule activators of ERSR in glioma cells. We performed a quantitative high-throughput screen (qHTS) against a collection of ~425,000 compounds and a comprehensive panel of orthogonal secondary assays was formulated for stringent compound validation. We identified novel activators of ERSR, including a compound with a 2,9-diazaspiro[5.5]undecane core, which depletes intracellular Ca^2+^ stores and induces apoptosis-mediated cell death in several cancer cell lines, including patient-derived and 3D cultures of glioma cells. This study demonstrates that our screening platform enables the identification and profiling of ERSR inducers with cytotoxic activity and advocates for characterization of these compound in *in vivo* models.

## Introduction

The endoplasmic reticulum (ER) is a multifunctional organelle involved in the synthesis, folding, storage and trafficking of proteins [1]. Protein storage and folding are aided by ER-resident molecular chaperones (mainly the glucose regulated protein 78, GRP78; also referred to as BiP) and high levels of Ca^2+^ in the ER lumen [2]. The ER is indeed a major site for Ca^2+^ storage and participates in fast Ca^2+^ responses underlying many signaling pathways. Disruption of ER homeostasis by physiological and pathological stimuli results in an accumulation of unfolded proteins (a condition known as ER stress) that triggers a complex cascade of events, referred to as the Endoplasmic Reticulum Stress Response (ERSR). These events are aimed at restoring homeostasis and involve an initial attenuation of global protein synthesis and a transcriptional remodeling to mobilize a cohort of stress response genes. The hallmark of ERSR activation is the increase in GRP78 expression, the main sensor of unfolded proteins and a key regulator of the ERSR. In resting conditions, GRP78 levels are low and GRP78 binds to and represses ERSR-activating proteins such as activating transcription factor 6 (ATF6), inositol requiring protein (IRE) 1α, PKR-like endoplasmic reticulum kinase (PERK), and the ER-associated caspase 4/12 (human/mouse, respectively). In the presence of unfolded proteins, conformational changes in GRP78 and active cycling of its ATPase domain, trigger the release of the aforementioned binding partners [3–5]. When stressing conditions are long-lasting or abnormally intense, activation of ERSR will lead to apoptosis. Apoptosis is achieved *via* three different mechanisms, including transcriptional activation of C/EBP homologous protein (CHOP), activation of c-Jun NH2-terminal kinase (JNK), and activation of ER-associated caspase 4/12. These mechanisms culminate in activation of terminal effector caspases and subsequent cell death [6–9].

Tumor cells display an elevated basal level of ERSR, which allows them to meet the ER demands of rapid cell division under a hostile environment (e.g. hypoxia and low pH) [10]. In fact, the protective effect of mildly elevated levels of ERSR has been correlated to chemotherapeutic tolerance [11–13]. Attempts to block elevated basal levels of ERSR in cancer cells, mainly by inhibiting IRE1α, and subsequent splicing of its target XBP1, have been reported as potential anticancer therapeutics [14, 15]. However, further stimulation of ERSR beyond a critical point is accompanied by enhanced cell death, suggesting that potent ERSR-inducing agents may possess antineoplastic potential [13, 16–18]. Small molecules that induce ERSR, including agents affecting ER Ca^2+^ homeostasis (e.g. thapsigargin and nonsteroidal anti-inflammatory drugs), protein folding or maturation (e.g. tunicamycin and brefeldin A), and misfolded protein removal (e.g. bortezomib), reportedly cause cytotoxicity [19]; however, these molecules are poor candidates for oncology applications due to suboptimal potency and selectivity, and/or poor bioavailability at the site of tumor formation.

**Fig 1:**
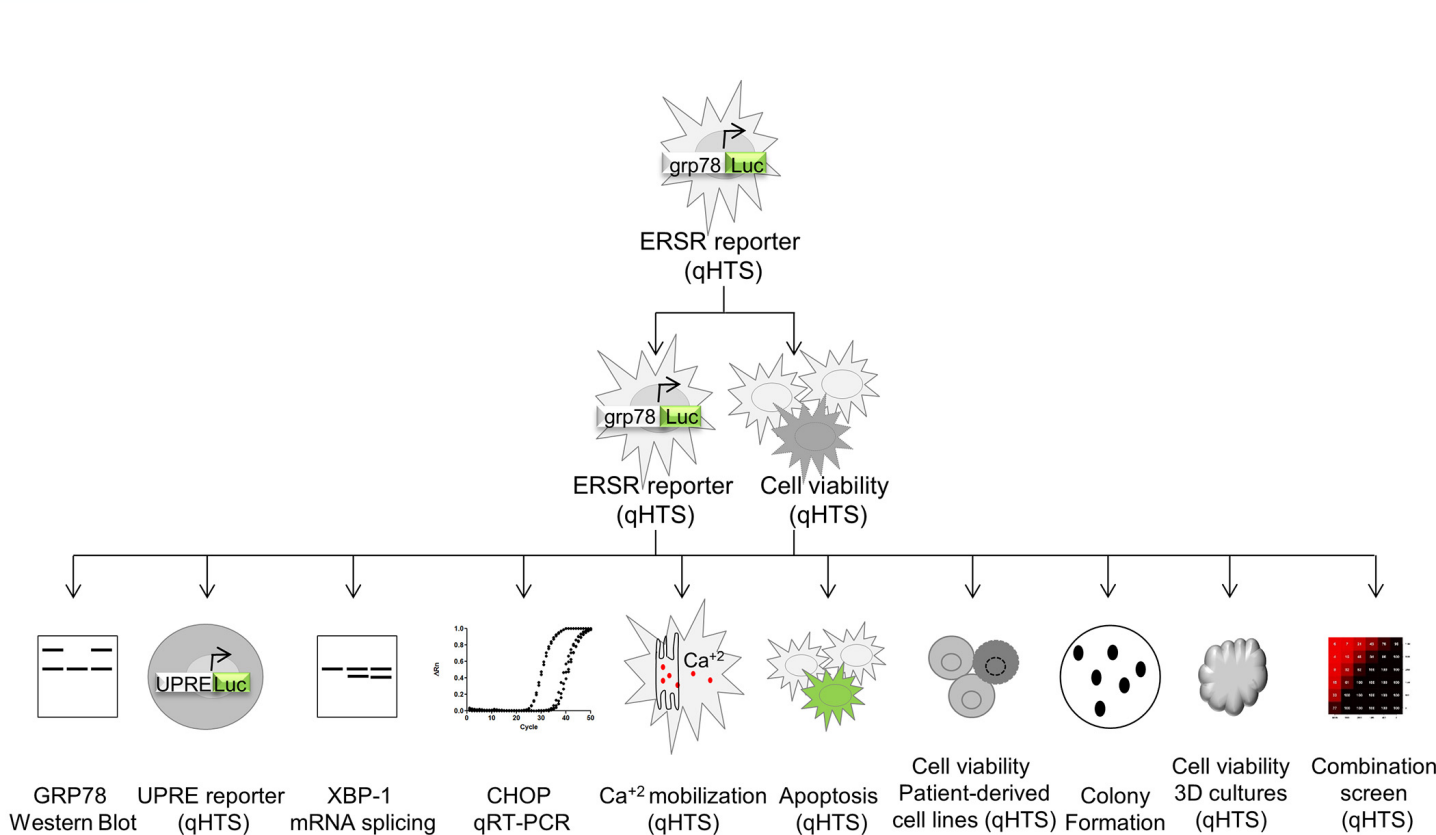
Schematic workflow for compound screening and triage. qHTS indicates assays performed in 1,536- and 384-well format. AC_50_ obtained in qHTS assays are summarized in Fig 2. Results of remaining low-throughput assays are summarized in Fig 3.

Our previous work has not only confirmed the ERSR as a relevant target to induce gliotoxicity but has also identified significant differences in the deployment of ERSR activation that render malignant glioma cells more susceptible to ERSR augmentation compared to normal astrocytes [20]. To further explore this therapeutic advantage, we sought to identify small molecules that activate ERSR and induce gliotoxicity. We implemented a cell-based quantitative high-throughput screen (qHTS) using a luciferase reporter that monitors GRP78 levels in human malignant glioma (U87-MG) cells. For triaging of the resulting screening hits, we devised a panel of secondary assays that validate reporter activation and inform on cytotoxicity potential and mechanism of action (Fig 1). We screened more than 425,000 unique compounds and identified several novel activators of ERSR that induce apoptosis-mediated cell death in multiple glioma cell lines, including U87-MG and patient derived lines, grown both in two-dimensional (2D) monolayers as well as physiologically relevant three-dimensional (3D) spheroid cultures. Furthermore, we synthesized a library of analogs around a hit compound containing an 2,9-diazaspiro[5.5]undecane core (compound **8**) and established a structure-activity relationship profile. We find that hit compounds also show dose-response cytotoxic effects in other non-glioma cancer cell lines indicating that these molecules have potentially broad anti-neoplastic activity. Finally, by pairwise combination screening, we show that compound **8** and its analogs synergize with hit compound **6** to reduce the viability of patient-derived glioma cell lines.

## Results

### Development, miniaturization, and qHTS of a grp78-luciferase assay

**Fig 2:**
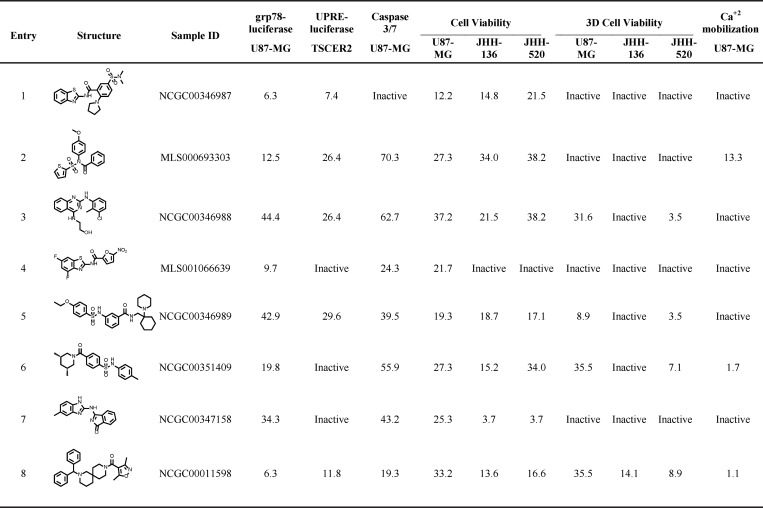
Chemical structure and AC50 (μM) of hit compounds in qHTS assays. For Ca2+ mobilization, values obtained in + EGTA conditions are displayed. Active compounds are considered those with a high quality CRC and efficacy. Efficacy cutoffs are as follow: grp78-luciferase and UPRE-luciferase, >40%; Caspase 3/7 and Ca2+ mobilization, >30%; all cell viability assays, >50%.

We have previously shown that ERSR activation as signaled by changes in the levels of GRP78 is a valuable predictor of cytotoxicity in glioma cells [20]. Therefore, we developed a cell-based assay that utilizes GRP78 as a biosensor to identify small molecule inducers of ERSR with associated cytotoxic properties. To this end, we engineered a stable U87-MG cell line to express a firefly luciferase reporter under the control of the GRP78 promoter (assay referred to herein as grp78-luciferase). The known ERSR activator thapsigargin elicited a concentration dependent increase on reporter activity with an EC_50_ of 11.4 nM (S1A Fig). Importantly, this increase in luciferase reporter expression is comparable to the increase in GRP78 native protein in U87-MG cells under the same treatment [21]. Since this assay could be affected by false negatives due to apoptosis-mediated cell death triggered by hit compounds, compound incubation time was optimized to 16 h, at which point luciferase induction achieves a maximal response of ~6-fold compared to vehicle-treated control. The ERSR activator tunicamycin also elicits an optimal reporter induction at 16 h (S1B Fig). qHTS provides concentration response curves (CRCs) of a chemical library directly from the primary screen and consequently has advantages over traditional screening paradigms in which each library member is tested at a single concentration [22, 23]. Therefore, we miniaturized the grp78-luciferase assay in 1,536-well format where a similar induction of ~6-fold and EC_50_ of 9.4 nM was observed with thapsigargin (S1C Fig and see S1 Supporting Information for additional details). To identify potential activators of ERSR, the grp78-luciferase assay was used in a robotic screen against a collection of 427,208 compounds contained in the NCGC chemical library, with 236 compounds exhibiting reporter activation (S1D Fig and S1 Supporting Information). Given that GRP78 activation leads to the deployment of the ERSR and potentially to cell death, we tested the activity of hit compounds in a cytotoxicity assay. To this end, we measured the viability of U87-MG cells after a 48 h compound exposure. The assay was run in 1,536-well format using the CellTiter-Glo (Promega) reagent, which quantifies cellular ATP levels as a proxy for viability. A total of 53 compounds were active in both the screening assay re-test and the CellTiter-Glo assays (S1E Fig). After we applied structural filters that eliminate electrophiles and other problematic compounds, a total of 8 compounds with favorable luciferase and cytotoxicity profiles (>100% and >50% efficacy, respectively) were chosen for further characterization (S2A and S2B Fig; Fig 2). To this end, we devised a panel of secondary assays designed to validate ERSR induction and cytotoxic activity (Fig 1).

### Effect of compounds on the activation of the three ERSR branches

**Fig 3:**
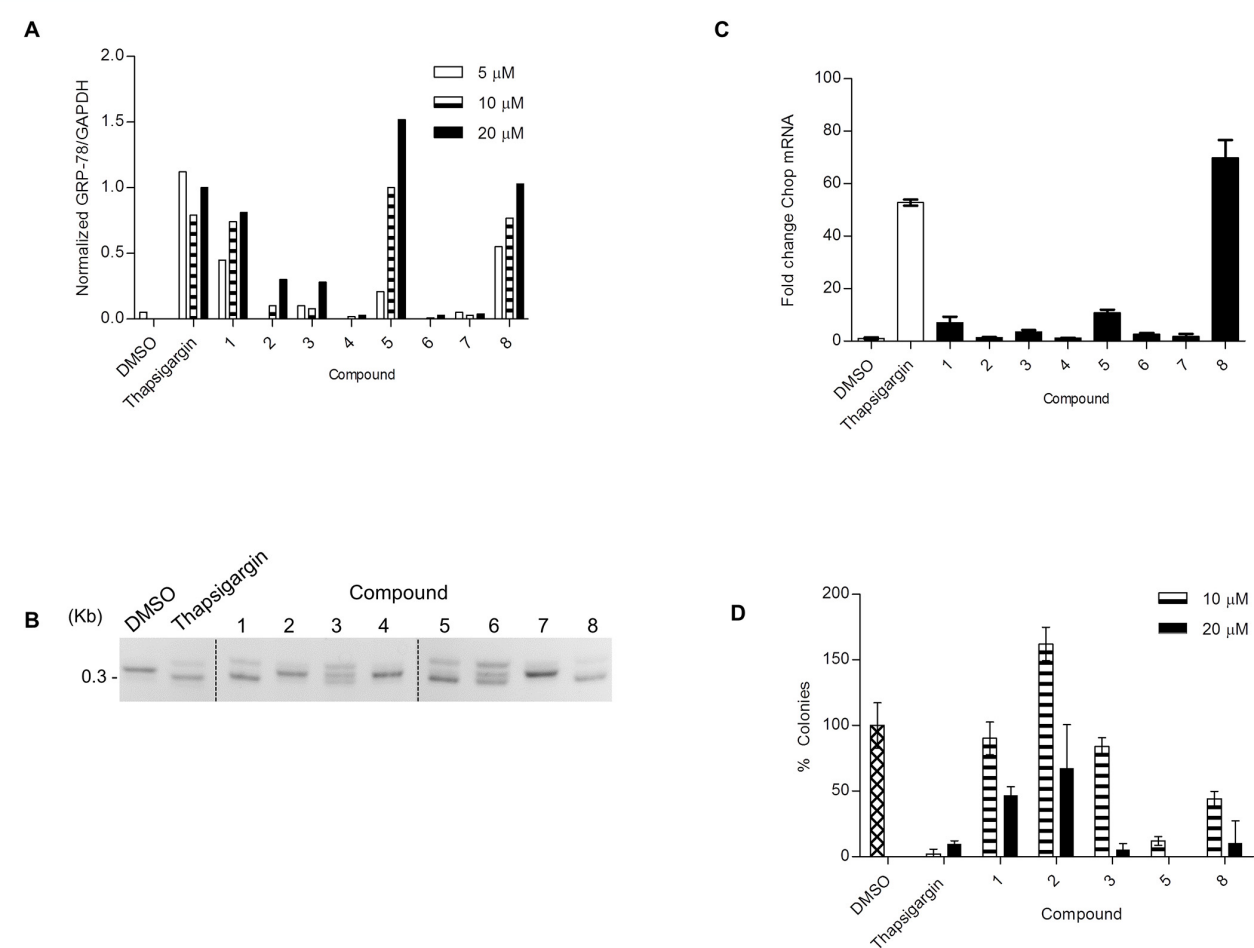
Secondary assays identify *bona fide* ERSR inducers with cytotoxicity activity. **(A)** U87-MG cells treated with the indicated compounds were analyzed by immunoblot for GRP78 expression. GAPDH levels were used as housekeeping controls. Data are represented as GRP78/GAPDH ratio for each compound and normalized to the GRP78/GAPDH ratio for 20 μM thapsigargin. See S3 Fig for blot images. **(B)** U87-MG cells were treated with DMSO, 1 μM thapsigargin or 20 μM of indicated compound for 16 h. XBP-1 mRNA splicing was monitored by RT-PCR using XBP-1-specific primers, which amplify a spliced or unspliced fragment of 304 or 326 bp, respectively. **(C)** U87-MG cells were treated with DMSO, 1 μM thapsigargin or 20 μM of indicated compound for 24 h. CHOP and housekeeping GAPDH mRNA levels were monitored by qRT-PCR. Data is plotted relative to the DMSO treated sample set to 1. Error bars indicate the SD of three replicates. **(D)** U87-MG cells treated with the indicated compounds were tested for their ability to form colonies. Data are presented as the number of colonies, normalized to vehicle DMSO. Error bars indicate SE with n = 3. See S5 Fig for representative images.

We first sought to confirm the effect of the 8 hit compounds on ERSR deployment. To determine whether hit compounds increase endogenous GRP78 levels, U87-MG cells were incubated with indicated compounds at concentrations of 5, 10, or 20 μM (concentration points shown to elicit significant response based on grp78-reporter CRCs) and subjected to western blotting using an anti-GRP78 antibody. As shown in Fig 3A, 5 out 8 compounds (compounds **1**, **2**, **3**, **5** and **8**) upregulate endogenous GRP78 to levels ≥30% higher than the induction achieved by treatment with 20 μM of the positive control thapsigargin. In fact, treatment of U87-MG cells with 10 and 20 μM concentrations of compounds **5** and **8** upregulated GRP78 protein to comparable levels as those achieved with thapsigargin (Fig 3A and S3 Fig).

In addition, we utilized a complementary cell-based assay that reports on transcriptional activation of ERSR signaling. Specifically, we engineered a human lymphoblastoid TSCER2 cell line to express a firefly luciferase reporter under the control of 5 copies of the unfolded protein response element (UPRE). The UPRE was originally identified as an ATF6 DNA binding element through *in vitro* binding site selection and was later shown to bind to spliced XBP-1. Importantly, it was reported to be strongly induced during the ER stress response [24–26]. The assay (referred to as UPRE-luciferase) was performed in 1,536-well format with each compound tested in dose response (S1 Supporting Information). We found that the same compounds that increased endogenous GRP78 protein levels also upregulated the UPR reporter (Fig 2; S2C Fig). Together, these results indicate that compounds **1**, **2**, **3**, **5** and **8** are able to inducethe ERSR response.We also investigated the effect of these compounds on two other components of the ERSR pathway. First, using an XBP1 mRNA splicing assay, which informs on activation of the IRE1α branch, we found that U87-MG cells treated with 20 μM of compounds **1**, **3**, **5, 6** and **8** for 16 h, have detectable levels of the XBP1 spliced transcript (Fig 3B). Second, we measured ATF4-mediated CHOP transcriptional induction to determine activation of the PERK branch [5]. We found that U87-MG cells treated with 20 μM of either compound **1** or **5** for 24 h display a 7–10 fold increase in CHOP transcript levels as measured by qRT-PCR (Fig 2C). Notably, compound **8** was the best-performing compound at increasing CHOP transcript levels (>60-fold compared to DMSO control).

### Effect of compounds on cytosolic Ca^2+^ levels

ER stress can be triggered by various stimuli, including disturbances in Ca^2+^ homeostasis. To identify activators that trigger ERSR by depleting intracellular Ca^2+^ stores, we employed a Ca^2+^ mobilization assay, using the fluorescent reporter Fluo-8 [27]. In this assay, the ionophore A23187 and thapsigargin, which functions by blocking the sarco/endoplasmic reticulum ATPase (SERCA), lead to an increase of cytosolic Ca^2+^. On the other hand, tunicamycin, an inhibitor of the UDP-N-acetylglucosamine-dolichol-phosphate N-acetylglucosamine-1-phosphate transferase (GPT) that causes accumulation of unfolded glycoproteins in the ER, does not affect cytosolic Ca^2+^ concentrations (S4A Fig). The assay was run in a 1,536-well format and compounds that exhibited a high quality CRC and >30% increase in Fluo-8 fluorescence were considered active. Of the 8 hits, compounds **6** and **8** strongly mobilized intracellular Ca^2+^ levels and compound **2** did as well but to a lesser extent (S2D Fig).

The elevated levels of cytosolic Ca^2+^ after compound treatment could in principle originate from the extracellular environment or intracellular stores like the ER or mitochondria. To distinguish between these two possibilities, we added 10 mM EGTA to the media to fully chelate extracellular Ca^2+^ before compound treatment (S4B Fig). Even in the presence of EGTA, compounds **2**, **6** and **8** were able to increase cytosolic Ca^2+^, indicating that they function to deplete intracellular Ca^2+^ stores (Fig 2 and S2E Fig).

### Effect of compounds on cell death

We sought to further investigate the cytotoxic effect of hit compounds. To verify apoptosis as the mechanism underlying the observed cytotoxicity, we tested compounds for their ability to activate key effector caspases using the Caspase-3/7 Glo detection system (Promega) (S1 Supporting Information). Compounds were tested as a dilution series and those that exhibited a high quality CRC and >30% increase in caspase 3/7 activation were considered active. Of the 8 hits, all the compounds showed significant caspase 3/7 activation except compound **1** (Fig 2; S2F Fig).

To assess the potential therapeutic use of the newly-discovered activators, we next tested their cytotoxic effects against two patient-derived suspension cell lines. The cell lines JHH-136 and JHH-520 were generated from grade 4 glioblastomas at the Johns Hopkins Medical Institute [28]. Specifically, we performed cell viability assays using the CellTiter-Glo reagent (S1 Supporting Information). Of the 8 compounds, only compound **4** failed to elicit a significant decrease in cell viability (Fig 2 and S2G and S2H Fig).

Next, we implemented a complementary clonogenic assay to examine the effects of compounds **1**, **2**, **3**, **5**, and **8** on cell survival and proliferation. Briefly, cells were plated at low density and proliferated to form colonies in the continuous presence of indicated compounds dozed at 10 and 20 μM. Importantly, the readout involves colony staining using crystal violet and counting as opposed to measuring global ATP levels as done in the CellTiter-Glo assay. As shown in Fig 3D and S5 Fig, at the highest concentration tested of 20 μM, compounds **1**, **3**, **5**, **8** markedly inhibited the colony forming capacity of U87-MG cells relative to DMSO-treated control. While compounds **1** and **3** were less effective at 10 μM, compounds **5** and **8** reduced colony numbers by more than 50% at that concentration. Compound **2** failed to significantly reduce colony numbers at either concentration tested.

Three-dimensional (3D) culture platforms are regarded in many ways as more physiologically relevant multicellular models than traditional monolayers. Cells maintained in 3D cultures have been reported to display altered sensitivities toward drugs [29, 30]. Hence, we tested the effect of the activators in a medium-throughput 3D spheroid culture assay. Briefly, cells were cultured in 384-well ultra-low attachment (ULA) plates for 3 days to allow the formation of aggregates. Of note, U87-MG cells formed a more compact spheroid compared to JHH-136 and JHH-520 cells (data not shown). Spheroids were treated with compounds for an additional 5 days and viability was measured using CellTiter-Glo (3D). Compounds **1**, **2**, **4** and **7** were inactive against all three cell lines. Compound **6** displayed activity in U87-MG and JHH-520 spheroids and compounds **3**, and **5** only in U87-MG spheroids. Compound **8** was the only one to elicit toxicity in all three cell lines (Fig 2 and S2I and S2K Fig).

### Structure-Activity Relationship Studies of an ERSR inducer

Our panel of secondary screens filtered out the majority of the compounds tested (Fig 2). Compounds **4**, **6** and **7** could embody weak ERSR activators or false positives of the grp78-luciferase assay, since they failed to induce activation of either one (compound **6**) or both (compound **4** and **7**) of the ERSR branches. Interestingly, they are able to induce apoptosis-mediated cell death, perhaps by a mechanism different from the apoptotic arm of the ERSR. When cells were treated with these three compounds, cell death was evident in monolayer cultures, and in the case of compound **6**, also in 3D cultures. On the other hand, compound **2** is likely a partial ERSR-inducing agent since it failed to induce CHOP mRNA as well as XBP-1 splicing. While it mobilizes intracellular Ca^+2^ and decreases viability of U87-MG cells in 2D CellTiter-Glo-based assays, compound **2** fails to reduce the colony forming capacity of U87-MG cells as well as the viability of spheroids of all three kinds. Based on these results, it is tempting to speculate that continuous exposure of U87-MG cells to compound **2** could induce ERSR but to levels that are still protective to cells. Alternatively, cells could develop resistance and overcome compound **2**-induced ERSR activation. Compounds **1**, **3**, and **5** activate all three branches of the ERSR and induce cell death in 2D cultures; however, they possess a limited ability to induce significant death when cells are cultured in 3D structures, making them less attractive for follow-up studies. The remaining compound **8**, on the other hand, met all the secondary screen criteria of a *bona fide* ERSR activator with intracellular mobilization Ca^+2^ potential and gliotoxic activity in all cell viability assays/formats tested (Fig 2).

We then resynthesized compound **8** and confirmed its ability to activate GRP78 and induce apoptosis in U87-MG cells (data not shown) and focused our medicinal chemistry optimization efforts on compound **8** to investigate systematic structure activity relationships.

**Fig 4:**
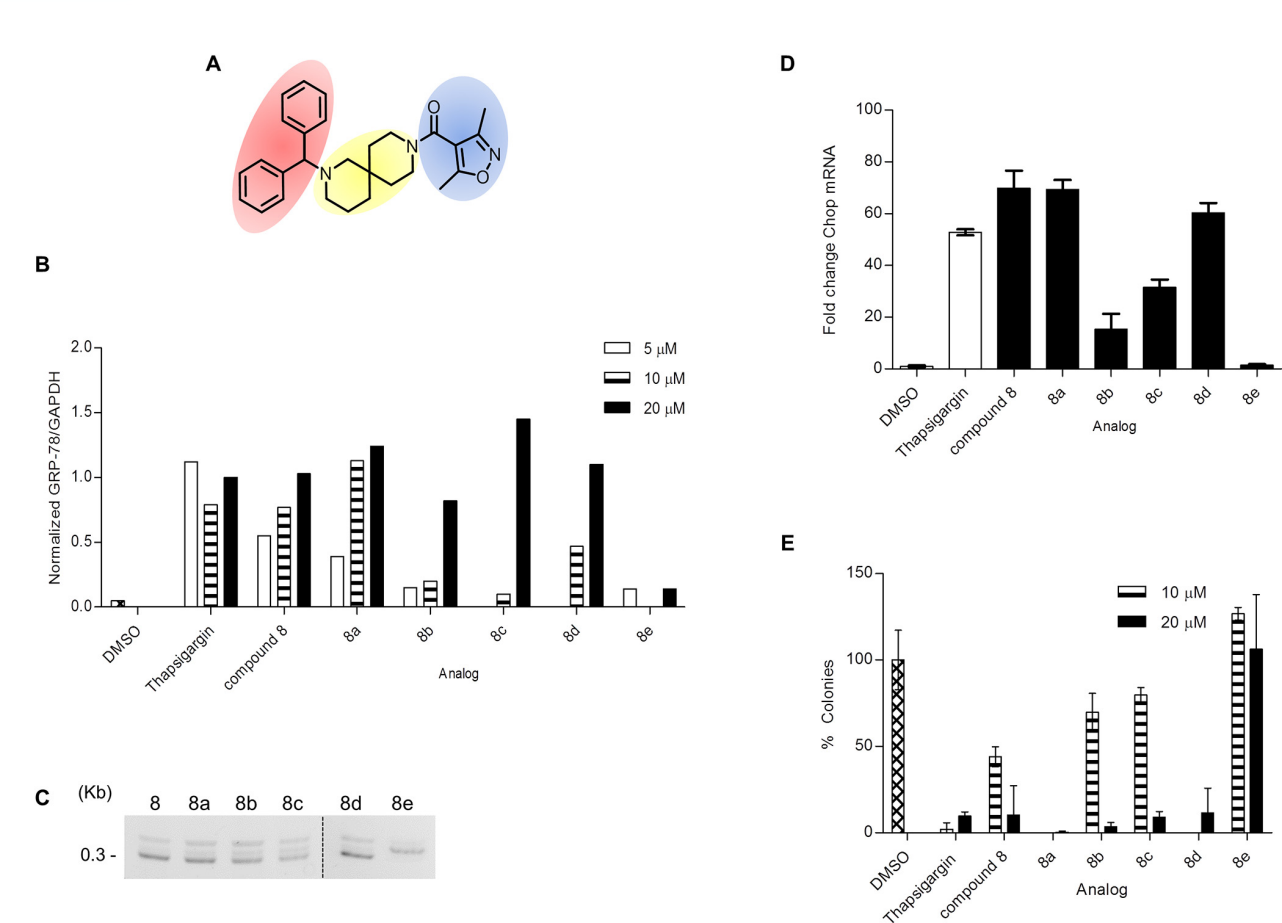
Structure-Activity Relationship for compound 8. **(A)** Structure of compound **8**. The 2,9-diazaspiro[5.5]undecane spirocyclic core, the diphenylmethyl group and the 3,5-dimethylisoxazole moiety are highlighted in yellow, red and blue, respectively. **(B)** U87-MG cells treated with the indicated analogs were analyzed by immunoblot for GRP78 expression. GAPDH levels were used as housekeeping controls. Data are represented as GRP78/GAPDH ratio for each compound and normalized to the GRP78/GAPDH ratio for 20 μM thapsigargin. See S3 Fig for blot images. **(C)** U87-MG cells were treated with DMSO, 1 μM thapsigargin or 20 μM of indicated analog for 16 h. XBP-1 mRNA splicing was monitored by RT-PCR using XBP-1-specific primers, which amplify a spliced or unspliced fragment of 304 or 326 bp, respectively. **(D)** U87-MG cells were treated with DMSO, 1 μM thapsigargin or 20 μM of indicated compound for 24 h. CHOP and housekeeping GAPDH mRNA levels were monitored by qRT-PCR. Data is plotted relative to the DMSO treated sample set to 1. Error bars indicate the SD of three replicates. **(E)** U87-MG cells treated with the indicated compounds were tested for their ability to form colonies. Data are represented as the number of colonies, normalized to vehicle DMSO. Error bars indicate SE with n = 3. Note that **8e** is an inactive control. Analogs **8a** and **8d** were tested only at 20 μM. See S7 Fig for representative images.

Briefly, the 2,9-diazaspiro[5.5]undecane spirocyclic core (Fig 4A, yellow circle) is key for the ERSR activity as measured by activity in the grp78-luciferase assay. Attempts to replace the core with several acyclic, monocyclic, bicyclic and fused ring systems resulted in a significant loss of potency (S2 Table). Similarly, the diphenylmethyl group on the left side of the molecule is essential to induce grp78 reporter activation (Fig 4A, red circle). Any structural modifications to the diphenylmethyl group triggered a complete loss of potency (S3 Table). Several structural changes to the 3,5-dimethylisoxazole moiety on the right side of the molecule were tolerated (Fig 4A, blue circle; S4 Table). However, the amide functionality proved to be strongly preferred as sulfonamide or methylene groups decreased the potency (S5 Table). Replacement of the dimethylisoxazole moiety with other heterocycles improved the potency but aryl/substituted aryl groups decreased the potency. Overall this chemotype exhibited very tight SAR with even subtle changes to the core region or diphenylmethyl groups resulting in a loss of potency. In summary, we synthesized and tested over 150 novel analogs (representative analogs are shown in S2–S5 Tables) around compound **8** with only a few of those showing comparable potency to the original hit.

### Selected analogs are *bona fide* ERSR inducers with cytotoxic activity in multiple cancer cell lines

**Fig 5:**
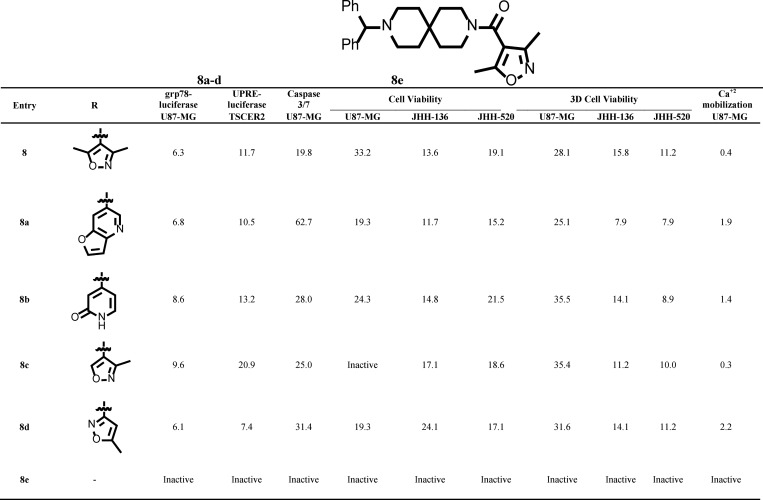
Chemical structure and AC50 (μM) of compound 8 analogs in qHTS assays. Efficacy cutoffs are the same as in Fig 2.

**Fig 6:**
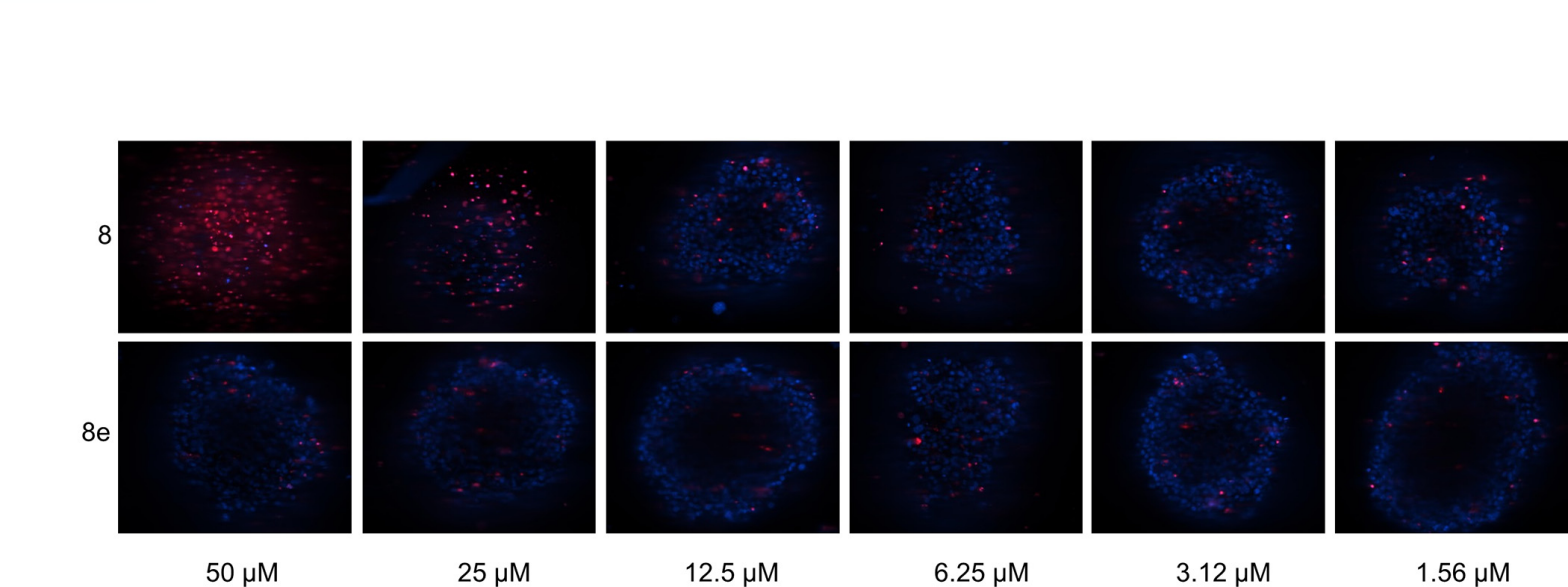
Compound 8 induces cell death in U87-MG spheroids. U87-MG spheroids were treated for 48 h with either compound **8** or inactive analog **8e** at the indicated concentrations. Spheroids were stained with Hoechst (blue) and propidium iodide (red) to mark nuclei and dead cells, respectively. For each spheroid, one single Z’ plane is shown.

**Fig 7:**
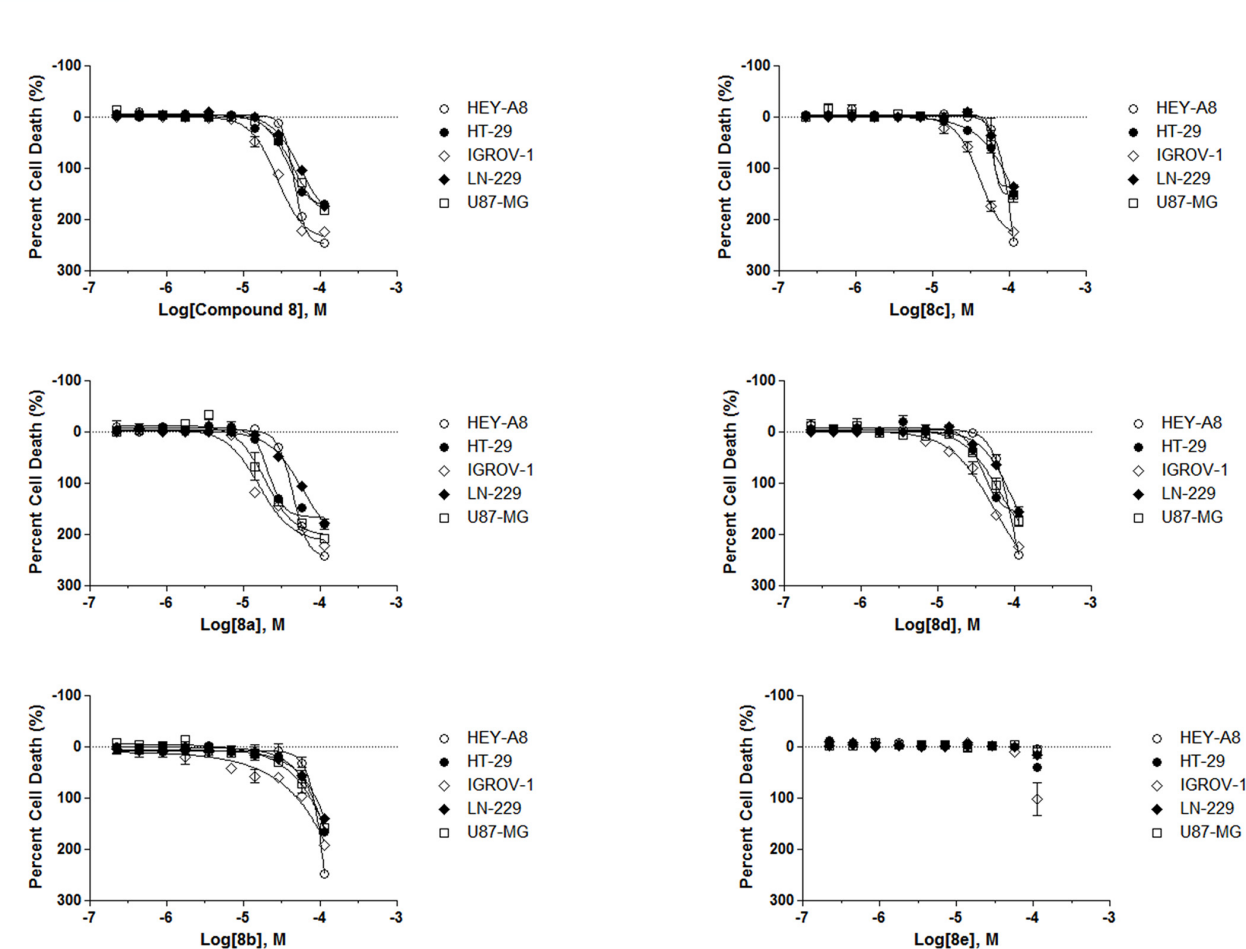
Compound 8 analogs reduce the viability of cancer cell lines. CellTiter-Glo viability assay of glioma (U87-MG and LN-229), ovarian (IGROV-1 and HEY-A8) and colon (HT-29) cancer cell lines treated for 48 h with compound **8** analogs.

We chose a set of 4 active analogs (**8a**, **8b**, **8c** and **8d**) and one inactive control molecule (**8e**) (Fig 5 and S6A Fig) for validation through our panel of secondary assays. We first look at the ability of these analogs to activate all three branches of the ERSR pathway. All active analogs increased endogenous GRP78 protein when tested at 20 μM. At lower doses however, only **8** and **8a** significantly increased GRP78 levels (Fig 4B). All active analogs induced ERSR activation as determined by the UPRE reporter assay, XBP-1 splicing and CHOP qRT-PCR assays (S6B Fig, Fig 3C and 3D, respectively) with compounds **8** and **8d** being the best performing molecules at increasing UPRE reporter levels and compounds **8** and **8a** at increasing CHOP transcript levels. Compound **8** was the most efficacious at mobilizing intracellular Ca^+2^ (S6C and S6D Fig). Importantly, the inactive analog **8e** did not induce ERSR nor mobilize intracellular Ca^+2^ stores. Second, we looked at the ability of these compounds to induce cell death. All active analogs induce apoptosis-mediated cell death in U87-MG, JHH-136 and JHH-520 cultures (S6E–S6H Fig). Colony forming assays indicated that at 20 μM, all active analogs were able to reduce the proliferating capacity of U87-MG cells (Fig 4E). When tested at 10 μM, the original hit was more efficacious than analogs **8c** and **8d** (analogs **8a** and **8d** were not tested at this concentration, Fig 4E). Notably, all analogs were able to decrease cell viability of 3D spheroids when incubated for 5 days (S6I–S6K Fig). U87-MG spheroids treated for 48 h with compound **8** had a higher population of dead cells, as measured by propidium iodide staining, compared to the inactive analog **8e** (Fig 6). Overall, none of the analogs showed significant potency improvements over the original hit compound. Fig 5 summarizes the AC_50_ values of compound **8** analogs in all qHTS assays. The cytotoxic effects for these compounds are not specific for glioma cell lines since treatment of ovarian (HEY-A8 and IGROV-1) and colon (HT-29) with compound **8** and analogs showed similar reduction in viability as seen in glioma (U87-MG and LN-299) cell lines (Fig 7).

### Selected compounds synergize to reduce cell viability

**Fig 8:**
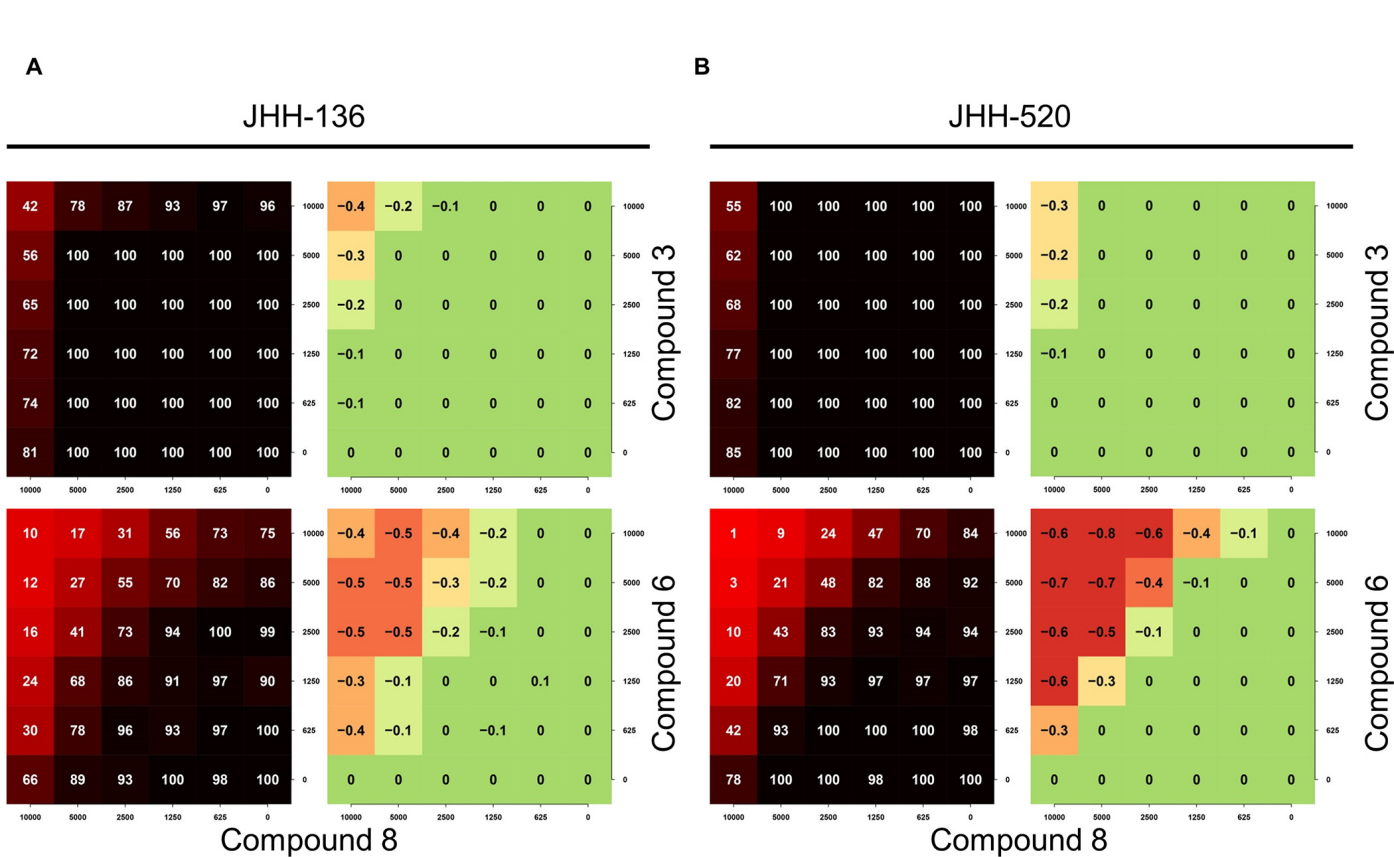
Compound 8 and 6 synergize to reduce the viability of patient-derived glioma cell lines. Combination (6 x 6) response profiles for compound **8**, **6**, and **3** in JHH-136 (A) and JHH-520 (B) cells. Each response profile is displayed as heatmaps of cell viability (percent response normalized to thapsigargin control; left panels) and DBSumNeg analysis (right panels).

Compounds that trigger ERSR via different mechanisms/targets could potentially synergize to induce cell death. We selected three validated ERSR inducers with cytotoxicity effects (compounds **8**, **8a** and **8c)**, a validated ERSR inducer with limited cytotoxicity (compound **3**), as well as a weak ERSR inducer (compound **6**) for combinatorial screening in JHH-136 and JHH-520 cell lines. We utilized a combination screening platform previously described [31, 32]. Briefly, we performed a pairwise 6x6 dose response matrix, where compounds were tested in a range of 0–10 μM (final concentration), to assess antagonistic, synergistic, or additive effects on cell viability. The highest dose of each compound (10 μM) was chosen as it is lower than the AC_50_ of each compound in cell viability assays (Fig 2). Viability response matrices were characterized using the Bliss model and summarized using the DBSumNeg metric [32]. For compound **8** and both analogs, we observed a strong synergism with compound **6** but not compound **3**, in both cell lines (Fig 8 and S8 Fig). Although compound **6** is a weak ERSR activator, it mobilizes intracellular Ca^2+^ to comparable levels as compound **8** does. It is tempting to speculate that a massive depletion of intracellular Ca^2+^ stores is causing the observed synergism. Importantly, no synergism was observed between compound **8** and its analogs, consistent with the idea that all of them work through the same mechanism/target (S9 Fig). No antagonism was observed for any of the combinations tested (Fig 8; S8 and S9 Figs).

## Discussion

We implemented an assay that quantitatively monitors GRP78 levels in human malignant glioma cells upon treatment with small molecules. We devised a compound triage strategy that included multiple quantitative high throughput as well as fixed-dose low throughput assays to enable the confident identification of small molecules with potent ERSR-inducing and apoptotic-mediated cytotoxic activities. Notably, a hit (compound **8**) with such properties was identified among our collection of over 425,000 unique chemical entities. Although further analyses are needed to fully understand the mechanism of action of this compound and its molecular target, it likely triggers ERSR by depleting intracellular Ca^2+^ stores. Our medicinal chemistry optimization efforts around compound **8** indicated that this chemotype exhibits very tight SAR and any significant changes to the molecule resulted in loss of potency. We describe here four top actives (analogs **8a**, **8b**, **8c** and **8d**), which along with the original hit compound, constitute a set of chemical tools worthy of *in vivo* ERSR-induced cytotoxicity studies.

To our knowledge, this is the first cell-based qHTS aimed to identify ERSR activators, *via* GRP78 induction, that induce apoptosis-mediated cell death. The work by Kudo et al. utilized a similar screening strategy to identify small molecules that induce GRP78 expression to protect neurons from ERSR-mediated apoptosis [33]. The authors identified BIX (Bip inducer X), which was shown to activate ER stress response elements through the ATF6 pathway but did not induce XBP-1 splicing nor CHOP expression. BIX was not included in our compound collection. In the recent report by Bi *et al*. the authors described the generation of a HeLa reporter cell line expressing β-lactamase under the control of the grp78 promoter [34]. The authors performed a pilot screen against the NIH Chemical Genomics Center Pharmaceutical Collection (NPC) of 2,800 drugs and identified 6 compounds that induce reporter expression. Four of those six drugs (6-thioguanine, primaquine, amlodipine and roxindole) tested negative in our primary screen; AMI-193 induced luciferase activity in our primary screen with an efficacy below 40% and was not included in follow up studies; the AMI-193-related drug, spiperone, induced reporter activity with an efficacy of ~40% but did not induce significant cell death of glioma cell lines. A different strategy was recently implemented to screen Chinese hamster ovary (CHO) K1 cells expressing luciferase constructs that individually reported on CHOP activation or IRE1α activity (via XBP1 mRNA splicing). This screen identified sulfonamidebenzamides as selective CHOP activators among a collection of 331,676 MLSMR compounds. One of these sulfonamidebenzamide derivatives showed antiproliferative activity against multiple cancer cell lines [35]. These compounds tested negative in our grp78-luciferase assay, consistent with GRP78 functioning upstream of CHOP and XBP1. However, the different cell types interrogated in these three studies could account for differential activity of identified ERSR activators.

The hits identified in this study provide excellent starting points not only for developing chemical probes but also to investigate the potential of combination therapy with other compounds, including ERSR stressors that function *via* alternative mechanisms. Indeed, we showed that compound **8** and analogs synergize with compound **6** to reduce the viability of patient-derived glioma cells. Other compounds of particular interest for combination studies are those that target autophagy. Autophagy and ERSR-mediated apoptosis are molecularly linked; thapsigargin and tunicamycin have both been shown to induce autophagy in several cell types [36–39]. Autophagy is currently considered a promising target for chemotherapy and targeting both pathways poses an attractive paradigm for oncology treatments [40–42].

## Experimental Procedures

### Cell lines and culture conditions

U87-MG, LN-229, and HT-29 cells were obtained from America Type Culture Collection, (ATCC #HTB-14, CRL-2611, and HTB-38, respectively). IGROV-1 and HEY-A8 were obtained from the NCI-60 panel of human cancer cell lines [43]. U87-MG, LN-229, HT-29, and HEY-A8 cells were cultured in Dulbecco's Modified Eagle Medium (DMEM; Life Technologies) supplemented with 10% fetal bovine serum (FBS; HyClone), 100 U/ml penicillin and 100 μg/ml streptomycin (referred to as 1% Pen/Strep; Life Technologies). IGROV-1 cells were cultured in RPMI-1640 (Life Technologies), supplemented with 2mM L-Glutamine (Life Technologies), 10% FBS and 1% Pen/Strep. Generation and characterization of JHH-136 and JHH-520 cell lines has been previously described [28]. Both cell lines were grown in suspension in Neurocult NS-A Stem Cell media (StemCell Technology), containing 20 ng/ml human EGF (Peprotech), 10 ng/ml human basic FGF (Peprotech) and 0.2% heparin (StemCell Technology). TSCER2 cells, derived from the human lymphoblast cell line TK6 [44], were cultured in RPMI 1640 medium (Life Technologies) supplemented with 5% FBS (Gemini Bio-Products), 1 mM sodium pyruvate (Life Technologies) and 1% Pen/Strep.

All cultures were maintained in a 37°C incubator with 5% CO_2_ and under a humidified atmosphere.

### Generation of grp78-luciferase and UPRE-luciferase stable cell lines

The pGL3-GRP78-(-132-+7)-Luc construct containing a firefly luciferase reporter gene under the control of the grp78 promoter was stably transfected into U87-MG cells. Transfection was performed using Lipofectamine 2000 reagent (Invitrogen) according to the manufacturer instructions. A clone that robustly and consistently expressed luciferase following treatment with either thapsigargin or tunicamycin was isolated and used for assay optimization. To this end, cells were seeded at 250,000 cells/well in 24 well dishes in DMEM+10% FBS. Compounds were added as DMSO solutions at the indicated concentrations and incubated for 8, 16, 24 or 48 hr. Luciferase activity was measured by adding a volume of Bright Glo (Promega) reagent as per manufacturer’s instructions.

The p5xUPRE-GL3 was stably transfected into TSCER2 cells [44] using a Gene Pulser apparatus (Bio-Rad, Hercules, CA) at 250 V and 950 μF. A clone that robustly and consistently expressed luciferase following treatment with either tunicamycin or 17-AAG was isolated and used for secondary screens.

### Colony Forming Assay

U87-MG cells were plated onto 6-well plates at a density of 1,000 cells/well in DMEM, supplemented with 10%FBS and 1% Pen/Strep and incubated O.N. at 37°C, 5%CO_2_, 95% RH. Cells were treated with either DMSO vehicle or compound solution at the indicated concentration (keeping a final concentration of 0.2% DMSO). Twelve days after compound treatment, cells were fixed, stained and counted as described [45].

### Western Blot

Assay is based on a previous method with modifications [20]. U87-MG cells were seeded (~3 × 10^5^ cells/well in 6-well plates) and treated with either vehicle DMSO, control thapsigargin or indicated compound at doses of 5, 10, or 20 μM for 16 h. Whole cell lysates were prepared using RIPA buffer (Cell Signaling) and protease inhibitor cocktail (Cell Signaling). Cell lysates were quantified using the Bio-Rad DC Protein Assay. 1 μg/μl of lysates were loaded on 4–12% gradient NuPAGE® Novex® Bis-Tris gels (Invitrogen) in MOPS SDS running buffer. Proteins were transferred to nitrocellulose membranes using the iBlot gel transfer system (Invitrogen) and blocked in TBST buffer (20 mM Tris pH 7.5, 150 mM NaCl, 0.05% Tween20, 5% BSA). Membranes were incubated with rabbit polyclonal anti-GRP78 primary antibody (# sc-13968, Santa Cruz Biotechnology) at a 1:1,000 dilution and mouse monoclonal anti-GAPDH antibody (# G8795, Sigma) at a 1:20,000 dilution. Either HRP- or Cy3/Cy5-conjugated secondary antibodies were used as follow: HRP-anti-rabbit IgG (# sc-2317, Santa Cruz Technology) or HRP-anti-mouse IgG (# sc-2031, Santa Cruz Biotechnology) were used at 1:2500 dilution and visualized with SuperSignal™ West Dura Chemiluminescent Substrate (Thermo Scientific) on a Bio-Rad Universal Hood II. ECL Plex-anti-rabbit IgG-Cy5 (# PA45012; GE Healthcare Life Sciences) or ECL Plex-anti-Mouse IgG-Cy3 (# PA43010, GE Healthcare Life Sciences) were used at 1:2,500 dilution and visualized using the Typhoon FLA 9500 (BPG1/532 nm/Cy3; LPR/635 nm/Cy5; GE). Protein quantification was performed using ImageQuant TL (GE) software.

### CHOP qRT-PCR and XBP-1 splicing assay

U87-MG cells were seeded (~3 × 10^5^ cells/well in 6-well plates) and treated with either DMSO, 1 μM thapsigargin or 20 μM of indicated compounds and incubated for either 24 (CHOP) or 16 h (XBP-1). RNA extraction and cDNA synthesis were performed using the TaqMan Gene Expression Cells-to-Ct Kit (TermoFisher Scientific) as per manufacturer’s instructions. Human CHOP and GAPDH transcripts were detected using the FAM-MGB Hs500358796_g1 and Human GAPD endogenous control (VIC-MGB, primer limited) probes, respectively (ThermoFisher Scientific) in a ViiA7 system (Applied Biosystems). XBP-1 splicing fragments were detected as described [34].

### Spheroid Imaging

U87-MG were cultured in DMEM, supplemented with 10% FBS and 1% P/S. Cells were plated onto 384-well, ultra-low attachment, spheroid microplates (Corning #3830) at a 750 cells/well/30 μl density using a Multidrop dispenser and spun down for 30 seconds at 1,000 rpm. Cells were incubated at 37°C, 5% CO_2_, under a humidified atmosphere for 3 days to allow spheroid formation (1 spheroid/well). Compounds were dissolved in growth media and 10 μl/well were added to a final concentration range of 156 nM to 50 μM (final assay contains 0.5% DMSO). To maintain the three dimensional structure, compounds were incubated for 48 h before imaging. Spheroids were stained with Hoechst 33342 (0.5 nM final) and PI (1 μg/ml final) for 2 h and imaged at 20X magnification on one Z’ plane using the InCell 6000 (GE) plate image reader.

### qHTS Assays

Detailed descriptions of all qHTS assays, primary screening, data analysis and associated protocols are included in S1 Supporting Information. S1 Table summarizes qHTS assay performance.

### General Chemistry Methods

Compound synthesis is described in S1 Supporting Information.

## Supporting Information

S1 Fig Development and characterization of a qHTS grp78-luciferase assay. (TIF)

S2 Fig Activity plots for 8 hit compounds in secondary qHTS assays. (TIF)

S3 Fig Western blot to assess endogenous GRP78 expression. (TIF)

S4 Fig An intracellularCa^2+^ mobilization assay. (TIF)

S5 Fig Colony forming assay. (TIF)

S6 Fig Activity plots for top compound 8 analogs in secondary validation qHTS assays. (TIF)

S7 Fig Colony forming assay. (TIF)

S8 Fig Analogs 8a and 8c synergize with compound 6 but not with compound 3, to reduce the viability of patient-derived glioma cell lines. (TIF)

S9 Fig Compound combinations that exhibit minimal to no synergism. (TIF)

S1 Supporting Information This file contains experimental procedures, supporting figure legends and references. (DOCX)

S1 Table Performance summary of qHTS assays. (PDF)

S2 Table SAR around the core. (PDF)

S3 Table SAR around the diphenylmethyl region. (PDF)

S4 Table SAR around the isoxazole region. (PDF)

S5 Table SAR around the amide region. (PDF)
