# Correction: A High-Throughput Screen Identifies 2,9-Diazaspiro[5.5]Undecanes as Inducers of the Endoplasmic Reticulum Stress Response with Cytotoxic Activity in 3D Glioma Cell Models

**DOI:** 10.1371/journal.pone.0166506

**Published:** 2016-11-08

**Authors:** Natalia J. Martinez, Ganesha Rai, Adam Yasgar, Wendy A. Lea, Hongmao Sun, Yuhong Wang, Diane K. Luci, Shyh-Ming Yang, Kana Nishihara, Shunichi Takeda, Mohiuddin, Irina Earnshaw, Tetsuya Okada, Kazutoshi Mori, Kelli Wilson, Gregory J. Riggins, Menghang Xia, Maurizio Grimaldi, Ajit Jadhav, David J. Maloney, Anton Simeonov

The eleventh author's name is listed incorrectly. The correct name is: Mohiuddin.
