# Supplementary figures and images for: A High-Throughput Screen Identifies 2,9-Diazaspiro[5.5]Undecanes as Inducers of the Endoplasmic Reticulum Stress Response with Cytotoxic Activity in 3D Glioma Cell Models
Source: PLoS One. 2016 Aug 29;11(8):e0161486. doi: 10.1371/journal.pone.0161486 (PMC5003374; doi:10.1371/journal.pone.0161486)

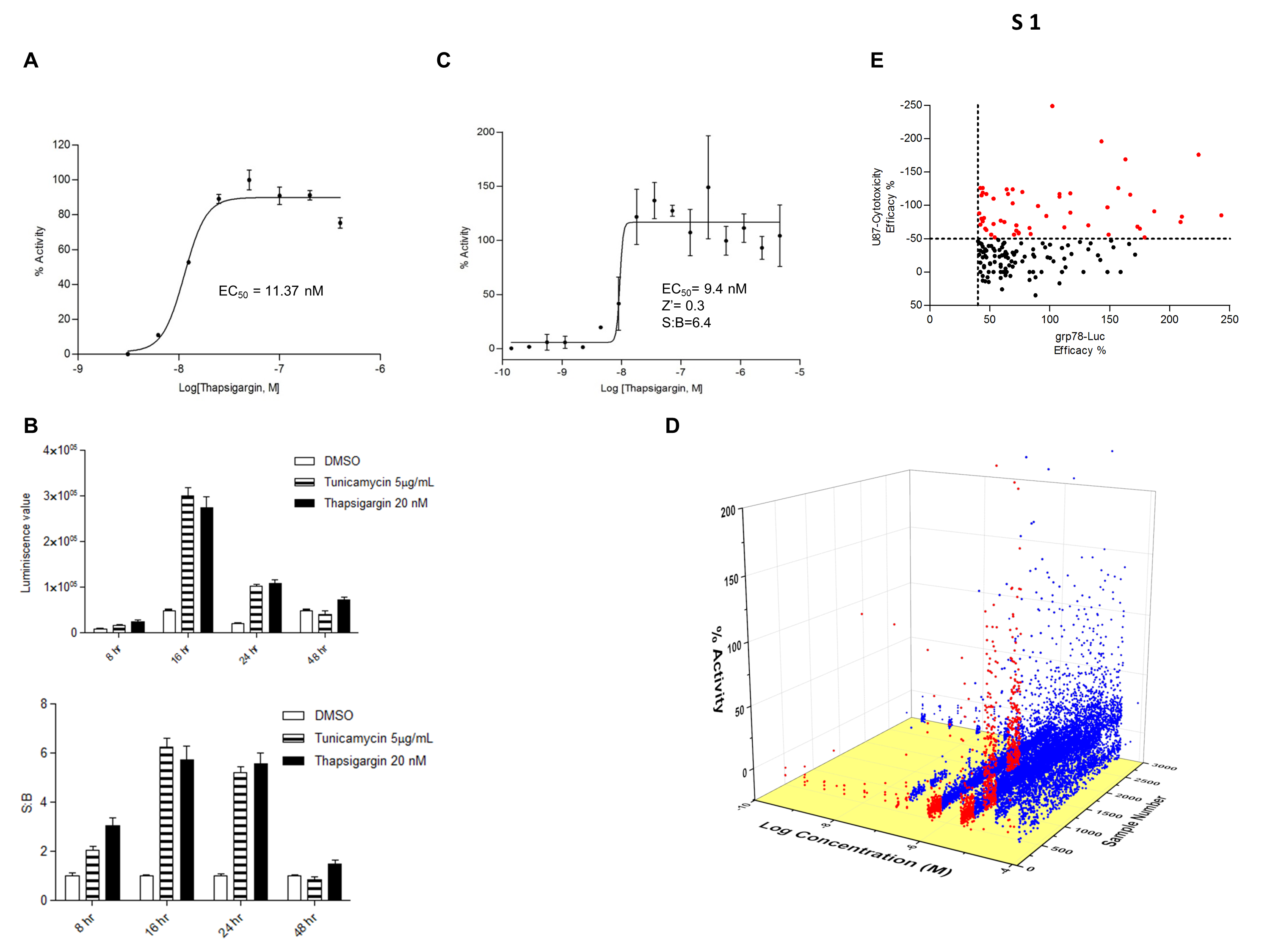

Supplement: S1 Fig — (TIF) [file pone.0161486.s001.TIF]

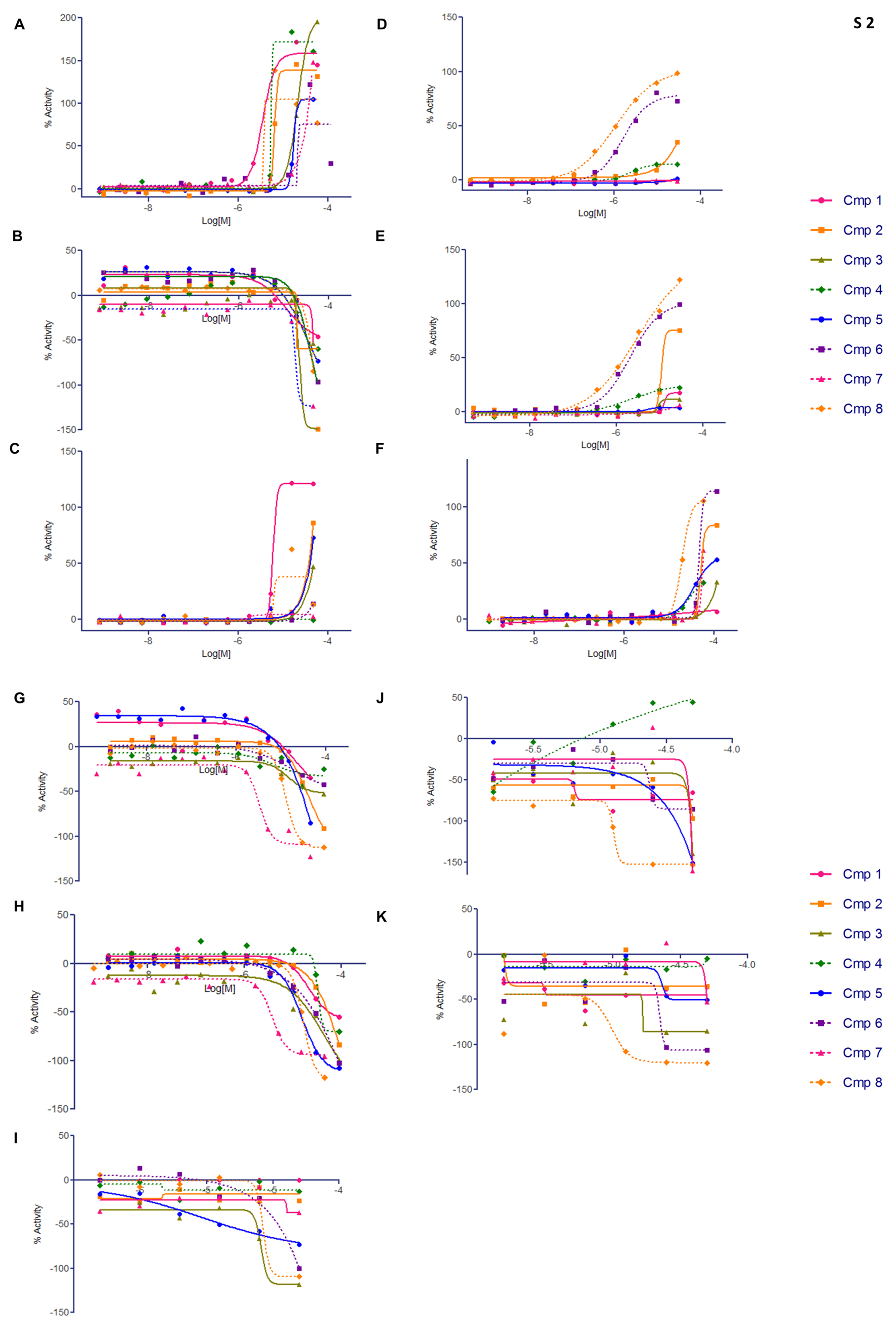

Supplement: S2 Fig — (TIF) [file pone.0161486.s002.TIF]

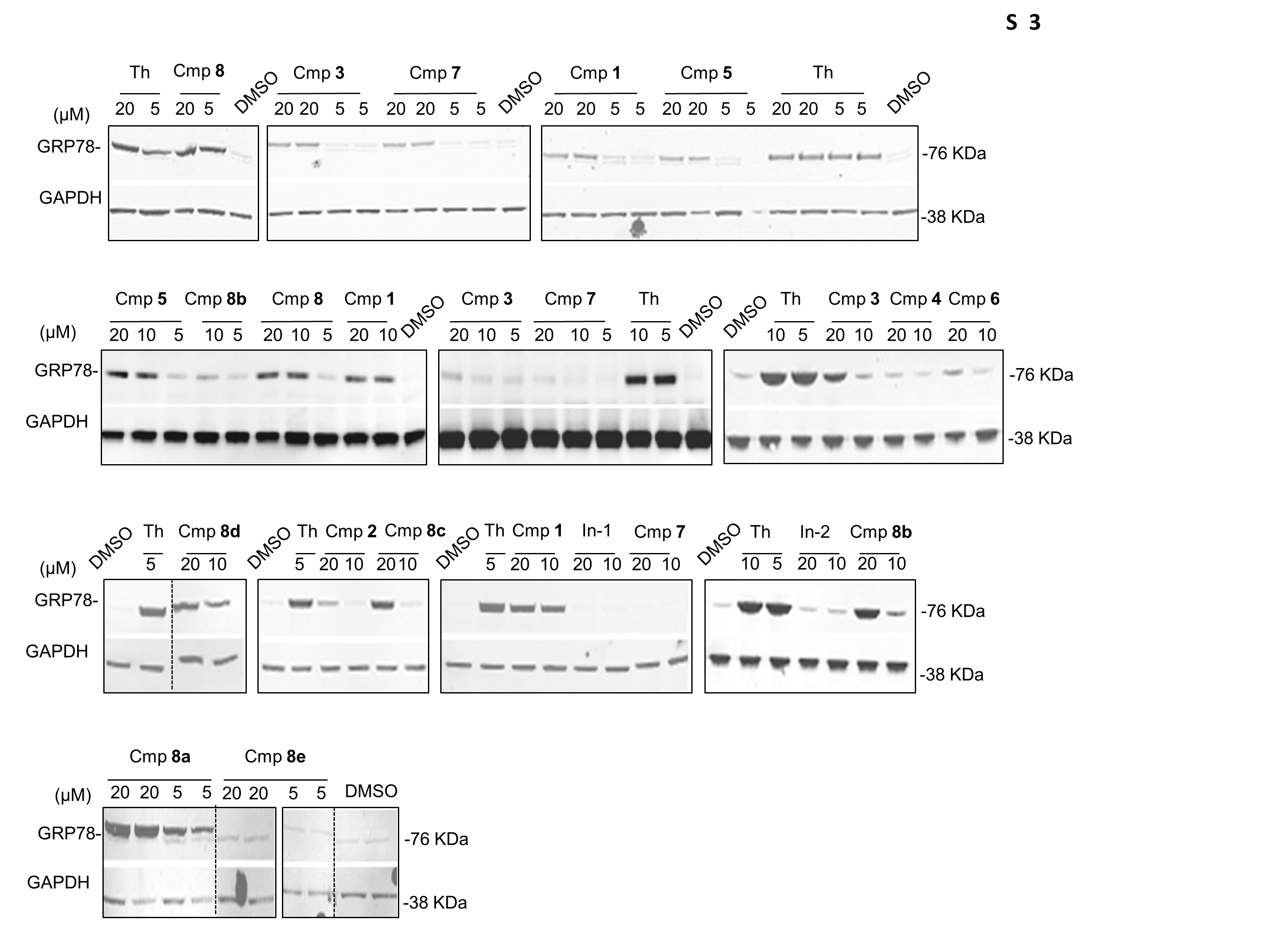

Supplement: S3 Fig — (TIF) [file pone.0161486.s003.TIF]

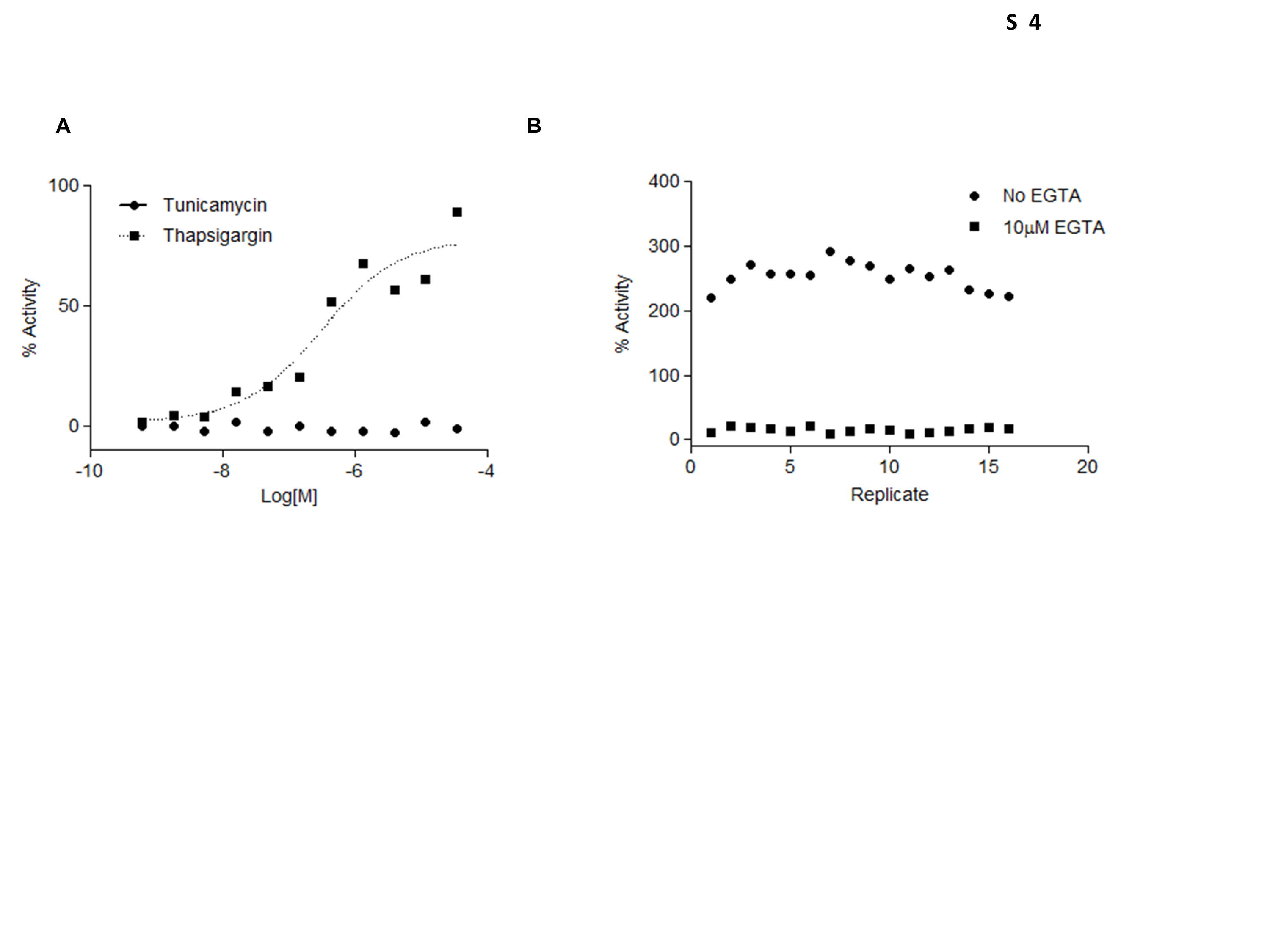

Supplement: S4 Fig — (TIF) [file pone.0161486.s004.TIF]

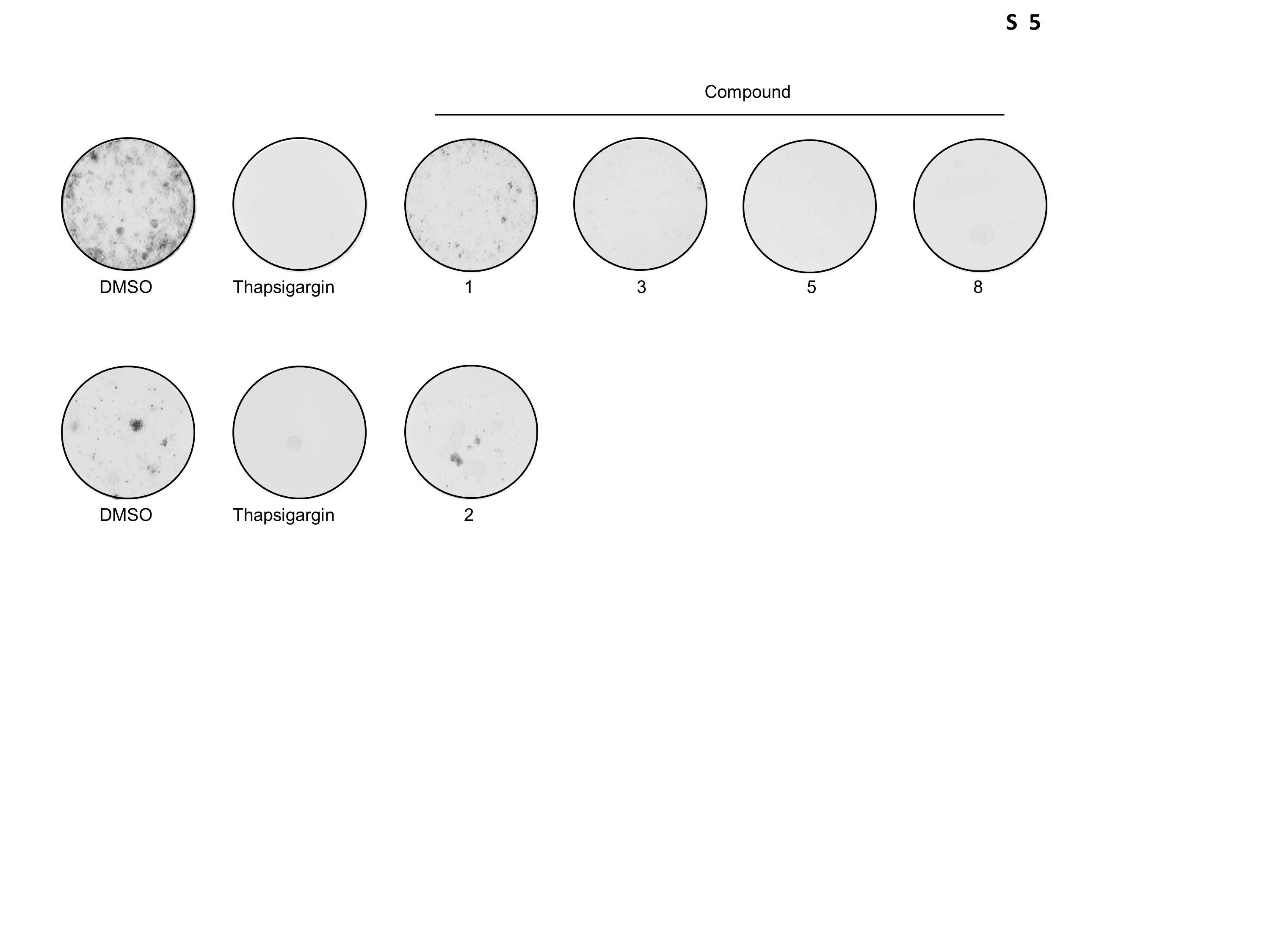

Supplement: S5 Fig — (TIF) [file pone.0161486.s005.TIF]

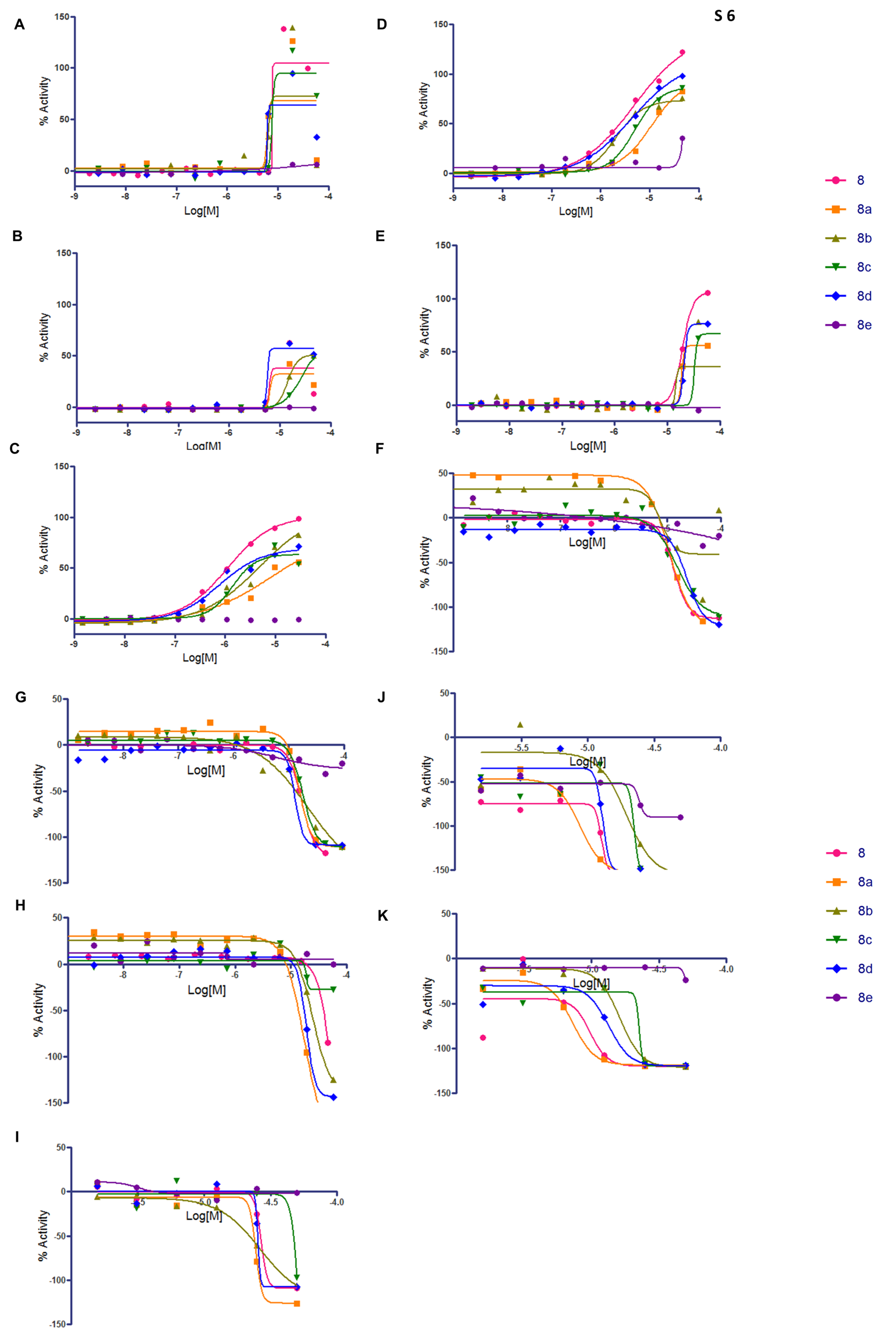

Supplement: S6 Fig — (TIF) [file pone.0161486.s006.tif]

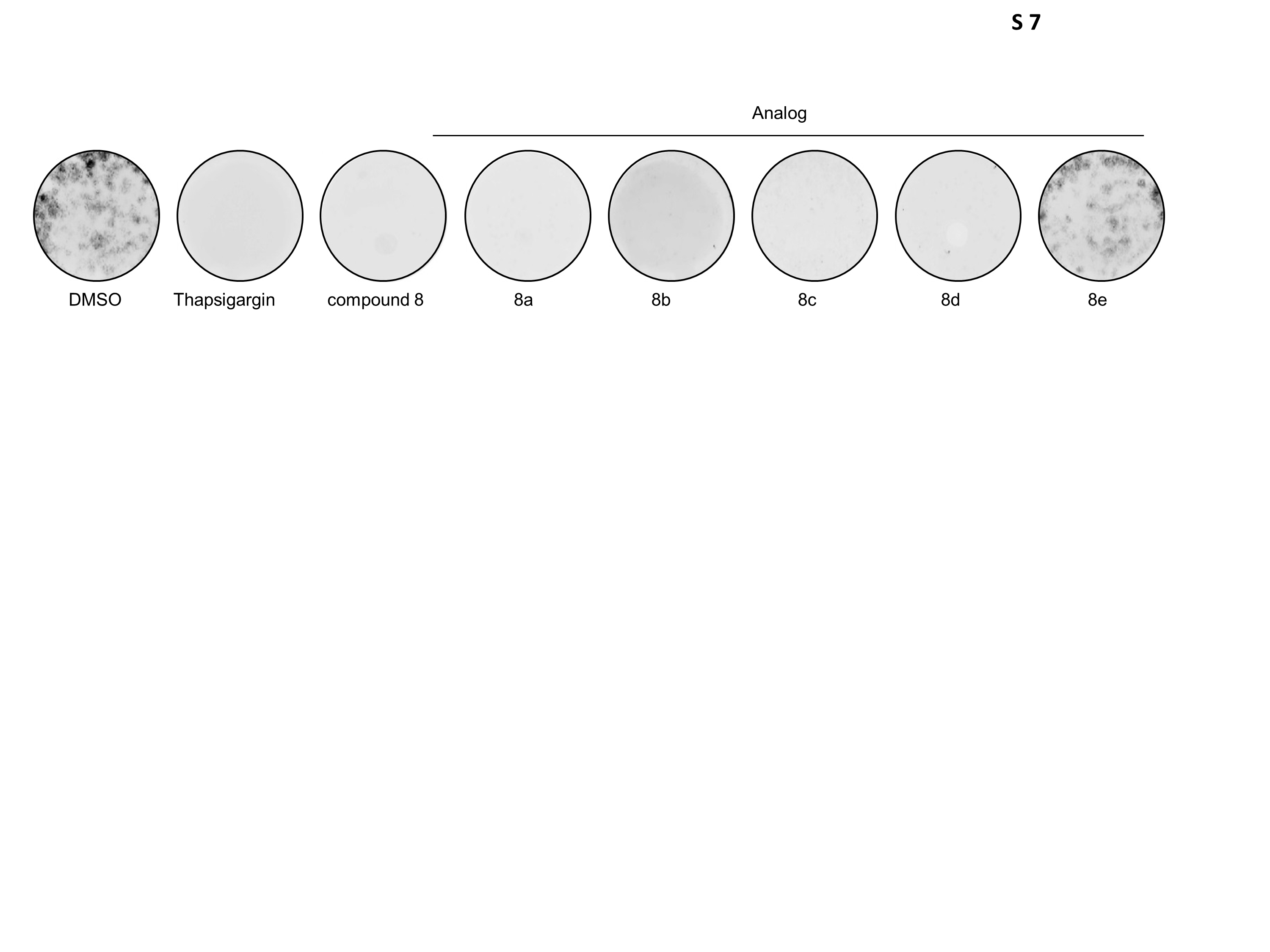

Supplement: S7 Fig — (TIF) [file pone.0161486.s007.TIF]

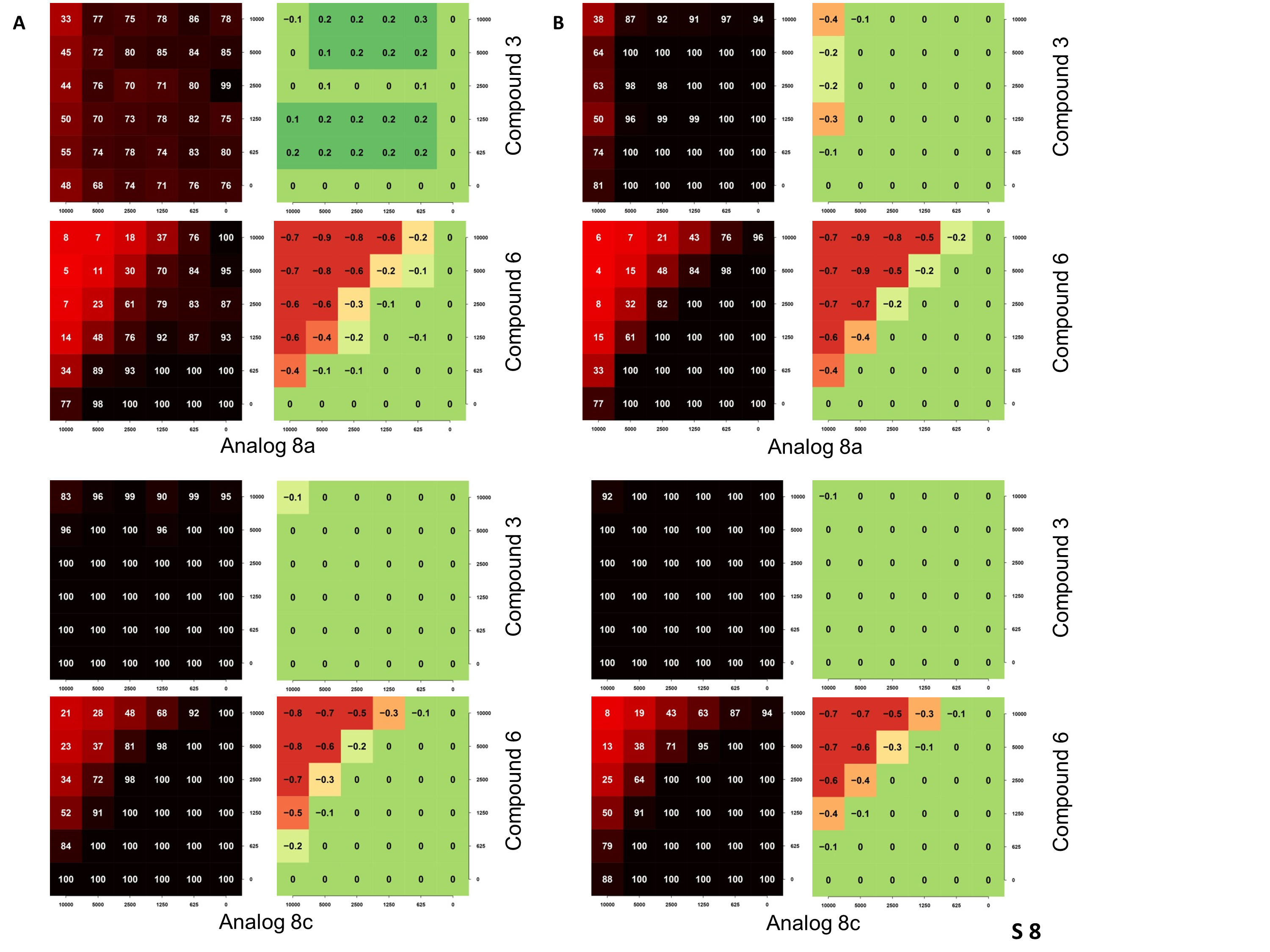

Supplement: S8 Fig — (TIF) [file pone.0161486.s008.TIF]

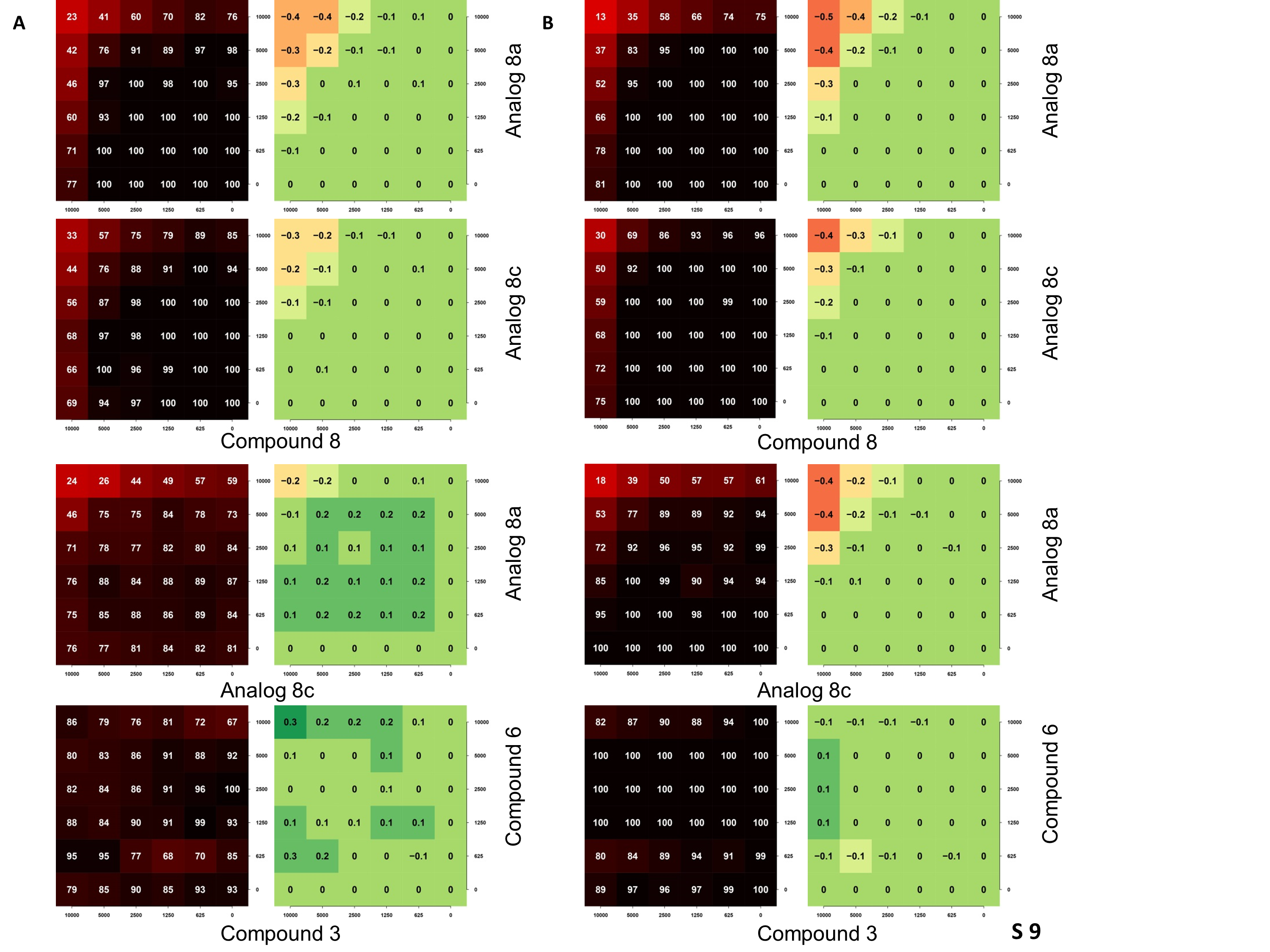

Supplement: S9 Fig — (TIF) [file pone.0161486.s009.TIF]
