# Supplementary material for: A High-Throughput Screen Identifies 2,9-Diazaspiro[5.5]Undecanes as Inducers of the Endoplasmic Reticulum Stress Response with Cytotoxic Activity in 3D Glioma Cell Models
Source: PLoS One. 2016 Aug 29;11(8):e0161486. doi: 10.1371/journal.pone.0161486 (PMC5003374; doi:10.1371/journal.pone.0161486)
Supplement: S1 Supporting Information — (DOCX) [file pone.0161486.s010.docx]

**Supplementary Information**

A high-throughput screen identifies 2,9-diazaspiro[5.5]undecanes as inducers of the endoplasmic reticulum stress response with cytotoxic activity in 3D glioma cell models

Natalia J. Martinez*^af^*, Ganesha Rai*^af^*, Adam Yasgar*^a^*, Wendy A. Lea*^a^*, Hongmao Sun*^a^*, Yuhong Wang*^a^*, Diane K. Luci*^a^*, Shyh-Ming Yang*^a^*, Kana Nishihara*^a,b^,* Sunichi Takeda*^d^*, Mohiuddin Sagor*^b^,* Irina Earnshaw*^b^,* Tetsuya Okada*^c^*, Kazutoshi Mori*^c^*, Kelli Wilson*^a,d^*, Gregory J. Riggins*^d^*, Menghang Xia*^a^*, Maurizio Grimaldi*^e^* , Ajit Jadhav*^a^*, David J. Maloney*^a^*^*^, and Anton Simeonov*^a*^*

*Corresponding authors ([asimeono@mail.nih.gov](mailto:asimeono@mail.nih.gov); [maloneyd@mail.nih.gov](mailto:maloneyd@mail.nih.gov))

This file includes:

Experimental procedures

Supplementary Figures S1-S9 legends

Supplementary Tables 1-5

References

*Experimental procedures*

*qHTS miniaturization and pilot screen*

After assay miniaturization, we performed a pilot qHTS, using an 8-point dilution series of the LOPAC^1280^ collection, with final compound concentrations ranging from 2.9 nM to 91.2 µM (PubChem AID 504836). The Z’-factor for the assay remained nearly constant throughout the experiment, with an average value of 0.58 ± 0.08, and a signal to background ratio (S:B) of 7.2 ± 0.4, indicating excellent assay performance. Intraplate titrations of the controls tunicamycin and thapsigargin exhibited robust performance, yielding average AC_50_ values of 17 nM and 21 nM, respectively.

*qHTS primary screen and secondary hit validation*

The grp78-luciferase assay was used in a robotic screen against a collection of 427,208 compounds contained in the NCGC chemical library. Each compound was tested as a 5-point dilution series, with concentrations ranging from 366 nM to 91.2 µM (PubChem AID 602332). The assay performance was adequately sensitive (S:B of 7.3 ± 1.0) and reproducible as indicated by statistical parameters for qHTS (Supplementary Table 1).

The primary screen identified 2,612 putative active compounds of which 240 exhibited an activity score >40, as defined by high-quality CRCs and luciferase signal six standard deviations above the population of neutral control wells, which corresponds to an efficacy of approximately 40% of the thapsigargin control. The remaining 2,372 compounds exhibited much weaker or noisier putative induction responses (activity score of <40 as defined by curve fit and efficacy) (S 1D). The overall hit rate was 0.056%, with hit compounds showing AC_50_ in the range of ~2 µM to 50 µM. From the above hits, we obtained 813 available compounds and subjected them to confirmatory re-testing in the grp78-luciferase assay. In addition, we tested them in a CellTiter Glo-based viability assay. Each compound was tested as an 11-point dilution series with concentrations ranging from 0.78 nM to 91.2 µM and both assays performed robustly (Supplementary Table 1). Of the 813 tested compounds, 156 confirmed in the grp78-luciferase assay and of those, 53 compounds were active in the cytotoxicity assay (as defined by high quality concentration curves and >50% efficacy) (S 1E).

grp78-luciferase assay:

U87-MG cells expressing grp78-luciferase were assayed in Opti-MEM® Reduced Serum Medium without Phenol Red (Invitrogen) supplemented with 5% FBS and 1% Pen/Strep. Cells were dispensed into 1,536-well, white, solid-bottom, TC-treated plates (Greiner Bio One) at 1,000 cells/5 µl/well using a Multidrop Combi dispenser (Thermo Scientific) and incubated (37°C, 5% CO_2_, under a humidified atmosphere) for 5 h. qHTS libraries (23 nl, final concentration range from 0.37 nM to 91.2 µM), normalization controls (neutral control DMSO; positive control thapsigargin at 1 µM), intraplate control titration thapsigargin (final concentration range 0.78 nM to 101 nM) and intraplate control titration tunicamycin (final concentration range 0.78 nM to 101 nM) were transferred using a pintool transfer station (Wako Automation). Cells were incubated overnight (37°C, 5% CO_2_, under a humidified atmosphere) and luminescence intensity was assayed by addition of 2.5 µl of Steady-Glo (Promega), followed by a 15 minute incubation at RT and measured using a ViewLux High-throughput CCD imager (Perkin Elmer) equipped with clear filters.

qHTS UPRE-luciferase assay:

Cells were dispensed at 1,000 cells/5 µl/well in 1,536-well white, solid bottom, TC-treated plates using a Multidrop dispenser, followed by addition of 23 nl of each compound (final range from 44.7 nM to 45.8 µM) into the assay plates using a pintool transfer station. Positive and neutral controls for normalization were 1 µM thapsigargin and DMSO, respectively. Tunicamycin and 17-AAG were used as intraplate control titrations. Assay plates were incubated for 6 h (37°C, 5% CO_2_, under a humidified atmosphere), followed by the addition of 5 µl of One-Glo reagent (Promega) into each well. The assay plates were subsequently incubated for 30 min at RT and luminescence intensity was measured using ViewLux reader.

qHTS Cell Viability Assay:

U87-MG, JHH-136 and JHH-520 cells were assayed in growth media at a density of either 1,000 (U87-MG) or 500 (JHH-136 and JHH-520) cells/ 5 μl/ well. Since JHH-136 and JHH-520 normally grow as spheres in suspension, we disrupted spheres by gentle pipetting till we obtained a single cell suspension, which was subsequently counted and plated accordingly. In all cases, cells were dispensed into 1,536-well, white, solid-bottom, TC-treated plates using a Multidrop dispenser and incubated at 37°C, 5% CO_2_, under a humidified atmosphere for 5 h. Compounds (23 nl, final concentration range from 0.775 nM to 91.2 µM) and controls were subsequently transferred via pintool. Positive and neutral controls for normalization were 5.7 µM thapsigargin and DMSO, respectively. Intraplate titration controls used were thapsigargin (titration range 656 pM to 401 nM for U87-MG cells and 33.6 nM to 4.6 µM for JHH-136 and JHH-520 cells). Cells were incubated for 48 h, followed by addition of 2.5 µl of CellTiter-Glo (Promega), then after a ~15 minute incubation at RT, samples were analyzed for luminescence intensity using a ViewLux reader.

For cell viability assays displayed in Figure 5, cells were assayed as above with the difference being that compounds were tested as 10-point dilutions series (range from 91.7 – 0.19 µM). Positive and neutral controls were thapsigargin (1 final µM) and DMSO, respectively.

qHTS 3D Cell Viability Assay:

U87-MG were cultured in DMEM, supplemented with 10% FBS and 1% P/S. JHH-136 and JHH-520 were grown in Neurocult NS-A containing 20 ng/ml human EGF, 10 ng/ml human basic FGF and 0.2% heparin. Cells were plated onto 384-well, ultra-low attachment, spheroid microplates (Corning) at a 750 cells/well/30 µl density using a Multidrop dispenser and spun down for 30 seconds at 1,000 rpm. Cells were incubated at 37°C, 5% CO_2_, under a humidified atmosphere for 3 days to allow spheroid formation (1 spheroid/well). Compounds were dissolved in growth media and 10 µl/well were added to a final concentration range of 156 nM to 50 µM (final assay contains 0.5% DMSO). Thapsigargin was used as positive control at a final assay concentration of 10 µM. Spheroids were incubated for 5 days, followed by addition of 30 µl of CellTiter-Glo 3D (Promega), then after a ~30 min incubation at RT and shacking at 500 rpm, samples were analyzed for luminescence intensity using a ViewLux reader.

qHTS Caspase 3/7 assay:

The effect of compounds and controls on caspase 3/7 activity was evaluated using the Caspase-Glo® 3/7 kit (Promega) according to the instructions provided by the manufacturer. Briefly, U87-MG cells were assayed in Opti-MEM® Reduced Serum Medium without Phenol Red supplemented with 5% FBS and 1% Pen/Strep at a density of 1,000 cells/4 or 5 µl/well. Cells were dispensed into 1,536-well, white, solid-bottom, TC-treated plates using a Multidrop dispenser and incubated at 37°C, 5% CO_2_, under a humidified atmosphere for 5 h. Compounds (23 nl, final concentration range from 0.78 nM to 114 µM) and positive control titration (thapsigargin, final concentration range 787 pM to 101 nM or staurosporine, final concentration range 2.8 nM to 45.6 µM) were transferred via pintool. Cells were incubated (37°C) 48 h, followed by addition of 3 µl of Caspase-Glo® 3/7 reagent, then after a 30 min incubation (RT) samples were analyzed for luminescence intensity using a ViewLux reader.

qHTS Ca^2+^ mobilization assays:

U87-MG cells were assayed in Opti-MEM® Reduced Serum Medium without Phenol Red supplemented with 1% FBS and 1% Pen/Strep at a density of 1,000 cells/4 μl/well. Cells were dispensed using a Multidrop dispenser onto a 1,536-well clear-bottom T/C black plate (Greiner Bio One) and incubated overnight (37°C, 5% CO_2_, under a humidified atmosphere). For assays where extracellular Ca^2+^ was chelated, 10 mM final of EGTA was added to cells before dispensing into plates. Three µl of Fluo-8 loading dye, prepared according to the manufacturer's instruction (Screen Quest™ Fluo-8 No Wash Calcium Kit; AAT Bioquest) was dispensed into each well. The assay plates were incubated at RT for ~15 minutes. Assay plates were then placed onto the FDSS7000 (Hamamatsu Photonics) kinetic fluorescence plate reader for measuring the changes of intracellular free calcium. The basal fluorescence signal was recorded for 10 s at 1 Hz followed by an addition of 23 nl of compounds and controls via pin tool transfer and 210 s continuously recording at 1 Hz. Compounds were tested as an 11-point dilution series at a final concentration range from 0.55 nM to 32.8 µM. Controls included A23187 (8.1 µM final), thapsigargin titration (final concentration range of 63.9 nM to 8.1 µM) and negative control tunicamycin titration (final concentration range of 30.7 nM to 3.9 µM).

qHTS data analysis and statistics

The primary screening data was analyzed using software developed internally in NIH Chemical Genomics Center. Data from each assay was normalized plate-wise to corresponding intra-plate controls as described previously ([1](#_ENREF_1)). The same controls were also used for the calculation of the Z’ factor for each assay. The Z’ factor, a measure of assay quality control, was determined by Z′ = 1 – (3 × SD_positive control_ + 3 × SD_neutral control_)/(Mean_positive control_ – Mean_neutral control_) where SD is the standard deviation ([2](#_ENREF_2)).

Hit compounds were analyzed *in silico* to remove compounds with poor development potential using three filters, namely, PAINS, Lilly Medchem filters (http://tripod.nih.gov/siphonify/) and the NCGC filter collection ([3](#_ENREF_3)). Percent activity was derived using in‐house software (http://tripod.nih.gov/curvefit/). Dose-response curves were classified as described previously ([4](#_ENREF_4)). Time-course fluorescence responses for the calcium mobilization assay, expressed in terms of fluorescent change over background, were utilized to derive percent activity curves using in-house software. All concentration–response curves were fitted and AC_50_ were calculated with the GraphPad Prism® software (GraphPad, San Diego, CA).

Matrix combination screening

Plating of compounds in matrix format using acoustic droplet ejection and numerical characterization of synergy, additivity and/or antagonism have been described previously ([5](#_ENREF_5), [6](#_ENREF_6)). Briefly, compounds were plated on a 6x6 dose combination matrix, with final assay concentration range of 0, 0.625, 1.25, 2.5, 5 and 10 µM. Compounds were acoustically dispensed (10 nl/well) using an ATS-100 (EDC Biosystems) onto 1,536-well, white, solid-bottom, TC-treated plates containing 2 µl of growth media. JHH-136 or JHH-520 cells were subsequently added to the plates (1,000 cells/3 µl /well) and incubated for 48 h at 37 °C with 5% CO_2_ under 85% humidity. Cell viability was determined by the addition of 3 μL of CellTiter-Glo into to each well. After a ~15 minute incubation at RT, samples were analyzed for luminescence intensity using a ViewLux reader. DMSO and thapsigargin (1 µM) were used as controls.

Combinations were characterized using the Bliss model and summarized using the DBSumNeg metric described in ([5](#_ENREF_5)).

*General Chemistry: Compound synthesis*

Preparative purification was run on a Waters semi-preparative HPLC system using a Phenomenex Luna C18 (5 micron, 30 x 75 mm) at a flow rate of 45 ml/min. A gradient of 10% to 50% acetonitrile in water over 8 minutes (each containing 0.1% trifluoroacetic acid) was used as a mobile phase during the purification. Fraction collection was triggered by UV detection (220 nm). Analytical analysis was performed on an Agilent LC/MS (Agilent Technologies, Santa Clara, CA). Method t1: A 7 min gradient of 4% to 100% Acetonitrile (containing 0.025% trifluoroacetic acid) in water (containing 0.05% trifluoroacetic acid) was used with an 8 minute run time at a flow rate of 1 ml/min. A Phenomenex Luna C18 column (3 micron, 3 x 75 mm) was used at a temperature of 50° C. Method t2: A 3 minute gradient of 4% to 100% Acetonitrile (containing 0.025% trifluoroacetic acid) in water (containing 0.05% trifluoroacetic acid) was used with a 4.5 minute run time at a flow rate of 1 ml/min. A Phenomenex Gemini Phenyl column (3 micron, 3 x 100 mm) was used at a temperature of 50° C. Method t3: Analysis was performed on an Agilent 1290 Infinity Series HPLC. UHPLC Long Gradient Equivalent 4% to 100% acetonitrile (0.05% trifluoroacetic acid) in water over 3.5 minutes run time of 4 minutes with a flow rate of 0.8 ml/min. Purity was determined using an Agilent Diode Array Detector for both Method t1, Method t2 and Method t3. Mass determination was performed using an Agilent 6130 mass spectrometer with electrospray ionization in the positive mode. ^1^H NMR spectra were recorded on Varian 400 MHz spectrometer. Chemical shifts are reported in ppm with undeuterated DMSO-d_6_ at 2.49 ppm as internal standard. High resolution mass spectrometry was recorded on Agilent 6210 Time-of-Flight LC/MS system. Confirmation of molecular formula was accomplished using electrospray ionization in the positive mode with the Agilent Masshunter software (version B.02).

A mixture of (bromomethylene)dibenzene (0.486 g, 1.966 mmol, 1 eq), tert-butyl 2,9-diazaspiro[5.5]undecane-9-carboxylate (0.5 g, 1.966 mmol, 1 eq), KI (0.326 g, 1.966 mmol, 1 eq), and TEA (0.548 ml, 3.93 mmol, 2 eq) in acetonitrile (4 ml) was stirred in a sealed tube for 6 h. The crude reaction mixture was directly loaded to an isco reverse phase column and purfied on ISCO flash system using water-acetonitrile as a mobile phase. The pure product obtained after removal of the solvent was taken up in DCM (10 ml) an added TFA (5 ml). Upon completion of the reaction, the reaction mixture was concentrated and dried under high vacum to get pure product as TFA salt (Yield - 64%).

A mixture of 2-benzhydryl-2,9-diazaspiro[5.5]undecane (0.05 g, 0.156 mmol, 1 eq), appropriate carboxylic acid (0.172 mmol, 1.1 eq) in DMF (0.5 ml) was added TEA (0.06 ml, 0.390 mmol, 2.5 eq) followed by a 50 % commercially available solution of Propylphosphonic anhydride solution (T_3_P^®^) in DMF (0.16 ml, 0.312 mmol, 2 eq) drop wise at rt . The reaction mixture was stirred at room temperature for 1 h then filtered through a bicarbonate cartridge. The filtrate was purified on a preparative HPLC to obtain pure product as TFA salt.

**4-(2-Benzhydryl-2,9-diazaspiro[5.5]undecane-9-carbonyl)pyridin-2(1H)-one (1)**: LC-MS Retention Time: t1 = 3.463 min and t2 = 2.436 min; ^1^H NMR (400 MHz, DMSO-d6) δ 11.72 (s, 1H), 7.44 – 7.37 (m, 5H), 7.28 (t, *J* = 7.5 Hz, 4H), 7.21 – 7.14 (m, 2H), 6.19 (dd, *J* = 1.6, 0.7 Hz, 1H), 6.07 (dd, *J* = 6.6, 1.6 Hz, 1H), 4.25 (s, 1H), 3.55 (dt, *J* = 12.3, 5.5 Hz, 1H), 3.33 – 3.20 (m, 2H), 3.07 – 2.95 (m, 1H), 2.37 – 1.97 (m, 4H), 1.62 – 1.21 (m, 8H); HRMS (ESI) *m/z* (M+Na)^+^ calcd. for C_28_H_31_N_3_NaO_2_, 464.2308; found 464.2315.

**(2-benzhydryl-2,9-diazaspiro[5.5]undecan-9-yl)(furo[3,2-b]pyridin-6-yl)methanone (2)**: LC-MS Retention Time: t1 = 4.625 min and t2 = 2.841 min; ^1^H NMR (400 MHz, DMSO-d6) δ 8.57 – 8.47 (m, 1H), 8.42 (d, J = 2.3 Hz, 1H), 8.09 (t, J = 1.3 Hz, 1H), 7.80 – 7.23 (m, 1oH), 7.23 – 7.15 (m, 1H), 5.56 (s, 1H), 4.22 (ddd, J = 16.1, 7.7, 4.7 Hz, 1H), 3.56 – 2.56 (m, 7H), 2.17 – 0.65 (m, 8H); HRMS (ESI) *m/z* (M+H)^+^ calcd. for C_30_H_32_N_3_O_2_, 466.2489; found 466.2506.

**(2-benzhydryl-2,9-diazaspiro[5.5]undecan-9-yl)(3,5-dimethylisoxazol-4-yl)methanone (3)**: LC-MS Retention Time: t1 = 4.225 min and t2 = 2.921 min; ^1^H NMR (400 MHz, DMSO-d6) δ 7.05 -7.81 (m, 10H), 5.56 (s, 1H), 3.71 -2.70 (m, 8H), 2.33 (s, 3H), 2.13 (s, 3H), 1.91 – 1.08 (m, 8H); HRMS (ESI) *m/z* (M+H)^+^ calcd. for C_28_H_34_N_3_O_2_, 444.2641; found 444.2646.

**(2-benzhydryl-2,9-diazaspiro[5.5]undecan-9-yl)(3-methylisoxazol-4-yl)methanone (4)**: LC-MS Retention Time: t3 = 1.83 min and t2 = 2.852 min; ^1^H NMR (400 MHz, DMSO-d6) δ 9.04 (s, 1H), 7.7 – 7.21 (m, 10H), 5.49 (s, 1H), 3.73 -2.62 (m, 8 H), 2.23 (s, 3H), 2.05 – 0.79 (m, 8H); HRMS (ESI) *m/z* (M+H)^+^ calcd. for C_27_H_32_N_3_O_2_, 430.2492; found 430.2489.

**(2-benzhydryl-2,9-diazaspiro[5.5]undecan-9-yl)(5-methylisoxazol-3-yl)methanone (5)**: LC-MS Retention Time: t1 = 4.308 min and t2 = 2.814 min; ^1^H NMR (400 MHz, DMSO-d6) δ 7.79 – 7.09 (m, 10H), 6.41 (s, 1H), 5.56 (s, 1H), 4.19 – 2.66 (m, 8H), 2.44 (s, 3H), 2.26 – 0.78 (m, 8H); HRMS (ESI) *m/z* (M+H)^+^ calcd. for C_27_H_32_N_3_O_2_, 430.2471; found 430.2489.

**(9-Benzhydryl-3,9-diazaspiro[5.5]undecan-3-yl)(3,5-dimethylisoxazol-4-yl)methanone (6)**: LC-MS Retention Time: t3 = 2.999 min and t2 = 3.107 min; ^1^H NMR (400 MHz, DMSO-d6) δ 7.76 – 7.59 (m, 4H), 7.48 (t, J = 7.5 Hz, 4H), 7.39 (dd, J = 8.4, 6.2 Hz, 2H), 4.16 – 2.99 (m, 8H), 2.35 (s, 3H), 2.16 (s, 3H), 2.03 – 1.23 (m, 8H); HRMS (ESI) *m/z* (M+H)^+^ calcd. for C_28_H_34_N_3_O_2_, 444.2641; found 444.2637.

*Supplementary Figure Legends*

**S 1: Development and characterization of a qHTS grp78-luciferase assay**. **(A)** Thapsigargin elicits a concentration-dependent induction of luciferase reporter at 24 hs. **(B)** Optimization of compound incubation time. Cells were treated with either tunicamycin or thapsigargin at the indicated concentrations and incubated for 8, 16, 24 or 48 hs. Raw luminescence values are shown on the top panel and signal to background (S:B) ratio (compound/DMSO) on the bottom panel. **(C)** Dose response curve for thapsigargin at 16 hs, in a miniaturized 1536-well assay format. **(D)** Activity plot of 2,612 compounds identified in the primary screen. Positioned in the front (•) are the 240 compounds that exhibited an activity score >40, while the remaining samples (•) are the 2,372 compounds that exhibited a weaker or noisier putative induction responses (activity score of <40). **(E)** grp78-luciferase and U87-MG cytotoxicity efficacy (%) correlation plot for 156 compounds that retested in grp78-luciferase assay (>40% efficacy). In red are 53 compounds that elicited >50% cytotoxicity in U87-MG cells.

**S 2: Activity plots for 8 hit compounds in secondary qHTS assays.** **(A)** grp78-luciferase induction after 16 h compound incubation. **(B)** U87-MG cell viability assays after 48 h compound incubation. **(C)** UPRE-luciferase assay after 6 h compound incubation. **(D, E)** Activity plots for 8 hit compounds in tertiary intracellular Ca^2+^ mobility assay in the absence (D) or presence of 10 mM EGTA (E). **(F)** Caspase 3/7 after 48 h compound incubation. **(G)** JHH-136 cell viability after 48 h compound incubation. **(H)** JHH-520 cell viability after 48 h compound incubation. **(I)** U87-MG 3D culture cell viability after 5 days compound incubation. **(J)** JHH-136 3D culture cell viability after 5 days compound incubation. **(K)** JHH-520 3D culture cell viability after 5 days compound incubation;

**S 3: Western blot to assess endogenous GRP78 expression.** Lysates from U87-MG cells treated with the indicated compounds were analyzed by immunoblot for GRP78 expression. Molecular weight markers (kDa) are shown on the right side. Note: 1) Cmp denotes compound and Th, thapsigargin; 2) In-1 and In-2 (ID NCGC00351063 and NCGC00119762, respectively) are compounds that tested inactive in the grp78-luciferase assay and were used as negative controls; and 2) Cmp1, 3, 5, 8 and 8b were tested in independent experiments.

**S 4:** **An intracellularCa^2+^ mobilization assay**. **(A)** Dose response curves for controls thapsigargin and tunicamycin. **(B)** Percent activity of A23187 (8 µM) in the presence or absence of 10mM EGTA to chelate extracellular Ca^2+^.

**S 5: Colony forming assay.** Representative images of U87-MG colonies after treatment with indicated hit compounds at a final concentration of 20 µM. Colonies formed in the presence of vehicle DMSO and 20 µM thapsigargin are shown for comparison on the left. Note that compound **2** was tested in an independent experiment with overall lower number of colonies.

**S 6: Activity plots for top compound 8 analogs in secondary validation qHTS assays.** **(A)** grp78-luciferase. **(B)** UPRE-luciferase. **(C, D)** Activity plots for 8 hit compounds in tertiary intracellular calcium mobility assay in the absence (C) or presence of 10 mM EGTA (D). **(E)** Caspase 3/7. **(F)** U87-MG cell viability. **(G)** JHH-136 cell viability. **(H)** JHH-520 cell viability. **(I)** U87-MG 3D culture cell viability. **(J)** JHH-136 3D culture cell viability. **(K)** JHH-520 3D culture cell viability.

**S 7: Colony forming assay.** Representative images of U87-MG colonies after treatment with indicated analogs at a final concentration of 20 µM. Colonies formed in the presence of vehicle DMSO and 20 µM thapsigargin are shown for comparison on the left.

**S 8: Analogs 8a and 8c synergize with compound 6 but not with compound 3, to reduce the viability of patient-derived glioma cell lines.** Combination (6 x 6) response profiles for analogs **8a and 8c**, and compound **6**, and **3** in JHH-136 (A) and JHH-520 (B) cells. Each response profile is displayed as heatmaps of cell viability (percent response normalized to thapsigargin control; left panels) and DBSumNeg analysis (right panels).

**S 9: Compound combinations that exhibit minimal to no synergism.** Combination (6 x 6) response profiles of JHH-136 (A) and JHH-520 (B) cells. Combinations between compound **8** and its analogs **8a** and **8b** exhibit minimal synergism. Similarly, combinations of compound **3** and **6** do not exhibit synergism. Each response profile is displayed as heatmaps of cell viability (percent response normalized to thapsigargin control; left panels) and DBSumNeg analysis (right panels).

*Supplementary Tables*

**Supplementary Table 1.**  Performance summary of qHTS assays.

| **qHTS Assay** | **Cell Line** | **Z'** | **Intraplate Control** | **AC_50_ [µM]** |
| --- | --- | --- | --- | --- |
| grp78-luciferase primary | U87-MG | 0.41 ± 0.21 | Thapsigargin | 0.051 ± 0.042 |
| grp78-luciferase secondary | U87-MG | 0.36 ± 0.19 | Thapsigargin | 0.066 ± 0.13 |
|  |  |  | Tunicamycin | 0.014 ± 0.006 |
| UPRE-luciferase | TSCER2 | 0.68 ± 0.06 | Thapsigargin | NA |
|  |  |  | Tunicamycin | 0.41 ± 0.007 |
|  |  |  | 17-AAG | 7.75 ± 0.38 |
| Caspase 3/7 | U87-MG | 0.78 ± 0.21 | Thapsigargin | 0.051 ± 0.005 |
| Cell viability | U87-MG | 0.48 ± 0.19 | Thapsigargin | 0.020 ± 0.022 |
|  |  |  | Tunicamycin | 0.016 ± 0.016 |
|  | JHH-136 | 0.43 ± 0.15 | Thapsigargin | 0.002 |
|  |  |  | Tunicamycin | 0.004 ± 0.0017 |
|  | JHH-520 | 0.51 ± 0.08 | Thapsigargin | 0.00025 ± 0.000173 |
|  |  |  | Tunicamycin | 0.0041 ± 0.001 |
| 3D Cell viability | U87-MG | 0.66 ± 0.09 | Thapsigargin | < 10 |
|  |  |  | Tunicamycin | < 1.6 |
|  | JHH-136 | 0.32 ± 0.03 | Thapsigargin | < 10 |
|  |  |  | Tunicamycin | < 1.6 |
|  | JHH-520 | 0.61 ± 0.12 | Thapsigargin | < 10 |
|  |  |  | Tunicamycin | < 1.6 |
| Calcium mobilization | U87-MG | 0.70 ± 0.15 | A23187 | NA |
| Matrix | JHH-136 | 0.64 ± 0.13 | Thapsigargin | 0.03 ± 0.02 |
|  | JHH-520 | 0.79 ± 0.05 | Thapsigargin | 0.0041 ± 0.004 |

**Supplementary Table 2.**  SAR around the core. Activity in grp78-luciferase assay is shown.

**Supplementary Table 3.**  SAR around the diphenylmethyl region. Activity in grp78-luciferase assay is shown.

**Supplementary Table 4.**  SAR around the isoxazole region. Activity in grp78-luciferase assay is shown.

**Supplementary Table 5.**  SAR around the amide region. Activity in grp78-luciferase assay is shown.

**References**

1. Seethala R, Zhang L. Handbook of drug screening. 2nd ed. New York: Informa Healthcare; 2009.

2. Zhang JH, Chung TD, Oldenburg KR. A Simple Statistical Parameter for Use in Evaluation and Validation of High Throughput Screening Assays. J Biomol Screen. 1999;4(2):67-73.

3. Jadhav A, Ferreira RS, Klumpp C, Mott BT, Austin CP, Inglese J, et al. Quantitative analyses of aggregation, autofluorescence, and reactivity artifacts in a screen for inhibitors of a thiol protease. J Med Chem. [Research Support, N.I.H., Extramural

Research Support, N.I.H., Intramural]. 2010 Jan 14;53(1):37-51.

4. Inglese J, Auld DS, Jadhav A, Johnson RL, Simeonov A, Yasgar A, et al. Quantitative high-throughput screening: a titration-based approach that efficiently identifies biological activities in large chemical libraries. P Natl Acad Sci USA. [Research Support, N.I.H., Extramural

Research Support, N.I.H., Intramural]. 2006 Aug 1;103(31):11473-8.

5. Griner LAM, Guha R, Shinn P, Young RM, Keller JM, Liu D, et al. High-throughput combinatorial screening identifies drugs that cooperate with ibrutinib to kill activated B-cell-like diffuse large B-cell lymphoma cells. P Natl Acad Sci USA. 2014 Feb 11;111(6):2349-54.

6. Mott BT, Eastman RT, Guha R, Sherlach KS, Siriwardana A, Shinn P, et al. High-throughput matrix screening identifies synergistic and antagonistic antimalarial drug combinations. Sci Rep. [Research Support, N.I.H., Extramural

Research Support, N.I.H., Intramural]. 2015;5:13891.
