# Supplementary material for: A High-Throughput Screen Identifies 2,9-Diazaspiro[5.5]Undecanes as Inducers of the Endoplasmic Reticulum Stress Response with Cytotoxic Activity in 3D Glioma Cell Models
Source: PLoS One. 2016 Aug 29;11(8):e0161486. doi: 10.1371/journal.pone.0161486 (PMC5003374; doi:10.1371/journal.pone.0161486)
Supplement: S1 Table — (PDF) [file pone.0161486.s011.pdf]

Supporting Table 1. Performance summary of qHTS assays.

qHTS Assay	Cell Line	Z'	Intraplate Control	AC_{50} [μM]
grp78-luciferase primary	U87-MG	0.41 ± 0.21	Thapsigargin	0.051 ± 0.042
grp78-luciferase secondary	U87-MG	0.36 ± 0.19	Thapsigargin	0.066 ± 0.13
			Tunicamycin	0.014 ± 0.006
UPRE-luciferase	TSCER2	0.68 ± 0.06	Thapsigargin	NA
			Tunicamycin	0.41 ± 0.007
			17-AAG	7.75 ± 0.38
Caspase 3/7	U87-MG	0.78 ± 0.21	Thapsigargin	0.051 ± 0.005
Cell viability	U87-MG	0.48 ± 0.19	Thapsigargin	0.020 ± 0.022
			Tunicamycin	0.016 ± 0.016
	JHH-136	0.43 ± 0.15	Thapsigargin	0.002
			Tunicamycin	0.004 ± 0.0017
	JHH-520	0.51 ± 0.08	Thapsigargin	0.00025 ± 0.000173
			Tunicamycin	0.0041 ± 0.001
3D Cell viability	U87-MG	0.66 ± 0.09	Thapsigargin	< 10
			Tunicamycin	< 1.6
	JHH-136	0.32 ± 0.03	Thapsigargin	< 10
			Tunicamycin	< 1.6
	JHH-520	0.61 ± 0.12	Thapsigargin	< 10
			Tunicamycin	< 1.6
Calcium mobilization	U87-MG	0.70 ± 0.15	A23187	NA
Matrix	JHH-136	0.64 ± 0.13	Thapsigargin	0.03 ± 0.02
	JHH-520	0.79 ± 0.05	Thapsigargin	0.0041 ± 0.004
