# Supplementary material for: A High-Throughput Screen Identifies 2,9-Diazaspiro[5.5]Undecanes as Inducers of the Endoplasmic Reticulum Stress Response with Cytotoxic Activity in 3D Glioma Cell Models
Source: PLoS One. 2016 Aug 29;11(8):e0161486. doi: 10.1371/journal.pone.0161486 (PMC5003374; doi:10.1371/journal.pone.0161486)
Supplement: S3 Table — (PDF) [file pone.0161486.s013.pdf]

Supporting Table 3. SAR around the diphenylmethyl region. Activity in grp78-luciferase assay is shown.

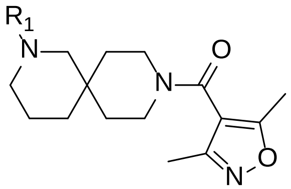

R_1	Activity	R_1	Activity
H	inactive	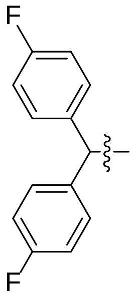	inactive
Me	inactive		
Ph	inactive		
Bn	inactive		
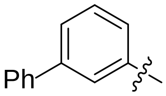	inactive	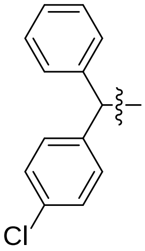	inactive
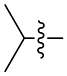	inactive		
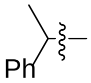	inactive		
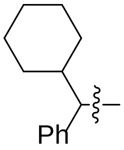	inactive	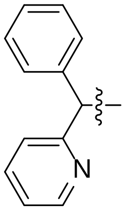	inactive
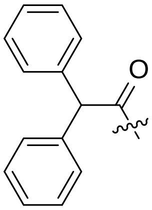	inactive	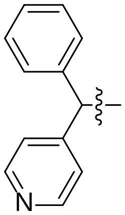	inactive
