# Supplementary material for: A High-Throughput Screen Identifies 2,9-Diazaspiro[5.5]Undecanes as Inducers of the Endoplasmic Reticulum Stress Response with Cytotoxic Activity in 3D Glioma Cell Models
Source: PLoS One. 2016 Aug 29;11(8):e0161486. doi: 10.1371/journal.pone.0161486 (PMC5003374; doi:10.1371/journal.pone.0161486)
Supplement: S5 Table — (PDF) [file pone.0161486.s015.pdf]

Supporting Table 5. SAR around the amide region. Activity in grp78-luciferase assay is shown.

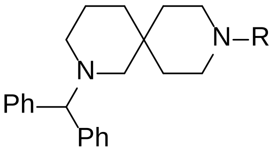

R	Activity
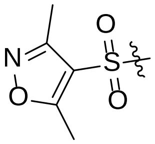	inactive
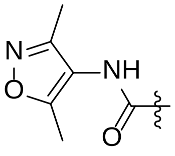	inactive
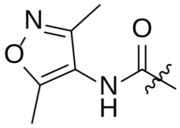	inactive
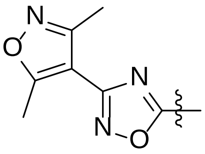	inactive
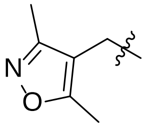	inactive
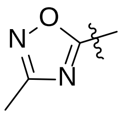	inactive
