# Supplementary material for: A High-Throughput Screen Identifies 2,9-Diazaspiro[5.5]Undecanes as Inducers of the Endoplasmic Reticulum Stress Response with Cytotoxic Activity in 3D Glioma Cell Models
Source: PLoS One. 2016 Aug 29;11(8):e0161486. doi: 10.1371/journal.pone.0161486 (PMC5003374; doi:10.1371/journal.pone.0161486)
Supplement: S2 Table — (PDF) [file pone.0161486.s012.pdf]

Supporting Table 2. SAR around the core. Activity in grp78-luciferase assay is shown.

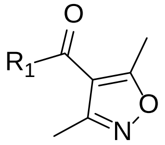

R_2 =

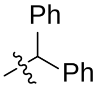

R_1	Activity	R_1	Activity		Activity
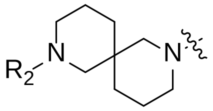	inactive	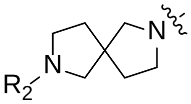	inactive	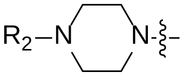	inactive
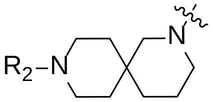	inactive	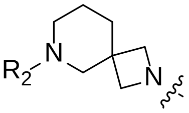	inactive	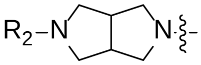	inactive
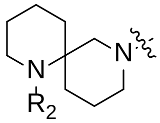	inactive	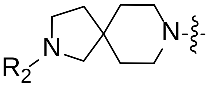	inactive	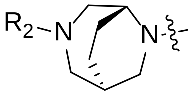	inactive
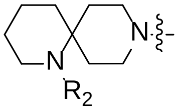	inactive	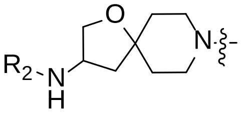	inactive	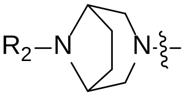	inactive
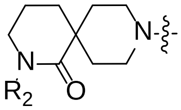	inactive	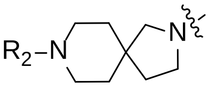	inactive	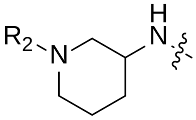	inactive
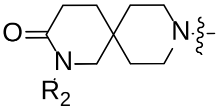	inactive	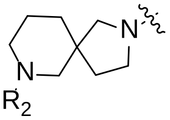	inactive	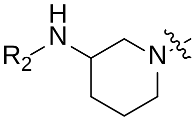	inactive
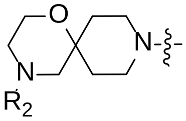	inactive	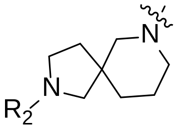	inactive	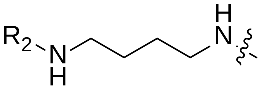	inactive
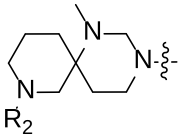	inactive	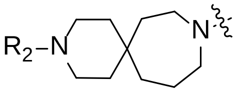	inactive	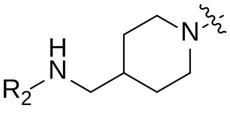	inactive
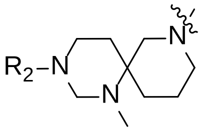	inactive	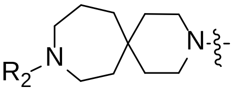	inactive	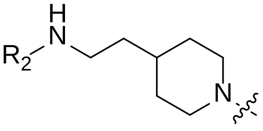	inactive
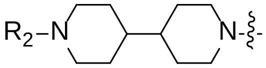	inactive	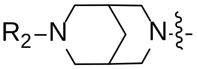	inactive	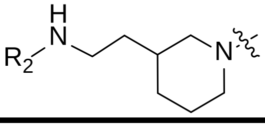	inactive
